# Rad51 paralogs and the risk of unselected breast cancer: A case-control study

**DOI:** 10.1371/journal.pone.0226976

**Published:** 2020-01-06

**Authors:** Peter Grešner, Ewa Jabłońska, Jolanta Gromadzińska

**Affiliations:** 1 Department of Toxicology and Carcinogenesis, Nofer Institute of Occupational Medicine, Lodz, Poland; 2 Department of Molecular Genetics and Epigenetics, Nofer Institute of Occupational Medicine, Lodz, Poland; 3 Department of Biological and Environmental Monitoring, Nofer Institute of Occupational Medicine, Lodz, Poland; CNR, ITALY

## Abstract

A case-control study was conducted in which we evaluated the association between genetic variability of DNA repair proteins belonging to the Rad51 family and breast cancer (BrC) risk. In the study, 132 female BrC cases and 189 healthy control females were genotyped for a total of 14 common single nucleotide polymorphisms (SNPs) within *Rad51* and *Xrcc3*. Moreover, our previously reported *Rad51C* genetic data were involved to explore the nonlinear interactions among SNPs within the three genes and effect of such interactions on BrC risk. The rare rs5030789 genotype (^-4601^AA) in *Rad51* was found to significantly decrease the BrC risk (OR = 0.5, 95% CI: 0.3–1.0, p<0.05). An interaction between this SNP, rs2619679 and rs2928140 (both in *Rad51*), was found to result in a two three-locus genotypes ^-4719^AA/^-4601^AA/^2972^CG and ^-4719^AT/^-4601^GA/^2972^CC, both of which were found to increase the risk of BrC (OR = 8.4, 95% CI: 1.8–38.6, p<0.0001), instead. Furthermore, rare *Rad51* rs1801320 (^135^CC) and heterozygous *Xrcc3* rs3212057 (^10343^GA) genotypes were found to respectively increase (OR = 10.6, 95% CI: 1.9–198, p<0.02) and decrease (OR = 0.0, 95% CI: 0.0-*NA*, p<0.05) the risk of BrC. Associations between these SNPs and BrC risk were further supported by outcomes of employed machine learning analyses. In *Xrcc3*, the ^4541^A/^9685^A haplotype was found to be significantly associated with reduced BrC risk (OR = 0.5; 95% CI: 0.3–0.9; p<0.05). Concluding, our study indicates a complex role of SNPs within *Rad51* (especially rs5030789) and *Xrcc3* in BrC, although their significance with respect to the disease needs to be further clarified.

## Introduction

Breast cancer (BrC) is known to be the most common malignancy among women, with nearly 1.7 million new cases and more than 520,000 deaths per year worldwide [1]. Its incidence is higher in North America and Western European countries contrary to Asian or African populations. Although not fully elucidated, mechanisms leading to BrC include a number of genetic and environmental factors, family history of the disease, multiparity, early menarche and late menopause [2].

Genetic association and GWAS studies provided valuable insights into genetic factors contributing to BrC risk. In addition to major BrC susceptibility genes including *BRCA1* and *BRCA2*, other high- (such as *TP53* and *PTEN*) and moderate- (*CHEK2*, *ATM*, *BRIP1*, *PALB2*, and *RAD51C*) penetrance susceptibility genes were found to play role in the onset of BrC [3–5]. Both *BRCA* genes together with the above-mentioned moderate-penetrance BrC susceptibility genes play their roles in homologous recombination (HR) DNA repair pathway involved in repair of DNA double strand breaks (DSB) [5–8]. It has been proposed that compromised capacity of the HR DNA repair system leads to increased accumulation of DNA damage, mutations and, hence, increased risk of malignancies [9–11].

The key component of the HR DNA DSB repair pathway is comprised by the Rad51 family proteins including Rad51, a crucial player in the whole HR DNA DSB repair machinery, and its five paralogs—Rad51B, Rad51C, Rad51D, Xrcc2, and Xrcc3. Paralogs interact with each other to form a hetero-tetrameric (Rad51B/Rad51C/Rad51D/Xrcc2; BCDX2) and hetero-dimeric (Rad51C/Xrcc3; CX3) complexes crucial for various processes involved in the HR DNA DSB repair machinery [12].

Rad51 (RecA homolog, *Escherichia coli*; 15q15.1) is a homolog of bacterial RecA protein forming a nucleoprotein filament on single-stranded DNA which in turn mediates the strand invasion and exchange between the damaged DNA sequence and its undamaged homologue thus facilitating the re-synthesis of damaged DNA region [13]. Xrcc3 (X-ray repair cross-complementing group 3; 14q32.3), on the other hand, has been shown to be crucial with respect to accumulation of Rad51 at sites of DNA DSB in the cell nucleus as well as to enzymatic resolution of the resultant cross-stranded structure (the Holiday junction) [13]. Rad51C (Rad51 homolog C, *S*. *cervisiae*; 17q25.1) seems to be required for RAD51/DNA nucleoprotein filament formation as it localizes to DNA DSB sites in early stages of HR [14], but it is also involved in DNA damage response and checkpoint activation [14], migration and resolution of Holiday junction [15], repair of interstrand cross-links [16] and stalled/collapsed replication forks as well as in antioxidant protection of mitochondrial genome [17,18]. Finally, its function as tumor suppressor and cancer susceptibility gene [5,19–21] has been proposed.

Most cancer association studies involving Rad51 were focused on two single nucleotide polymorphisms (SNPs) localized in the 5′ untranslated region (5’UTR) of exon 1 of the gene: rs1801320 (c.−98G>C; ^135^G/C) and rs1801321 (c.−61G>T; ^172^G/T). Both these SNPs were reported to be associated with altered gene transcription [22,23]. Large meta-analyses have shown that ^135^C allele increases the general risk as well as the risk of BrC, with a distinct dose-dependent effect [10,24]. The ^172^T allele-containing genotypes, on the other-hand, were found to be associated with some 25% reduction of general odds of cancer compared to the ^172^GG wild-type one [10]. Nevertheless, association between ^172^T allele and the risk of BrC seems to be much more complex, as only limited number of studies are available so far, suggesting both increase [25,26] as well as decrease [27,28] of the disease risk being associated with the allele, not allowing us thus to draw any final conclusion.

In the case of Xrcc3, rs861539 (c.722C>T; ^241^Thr/Met) in exon 8 is the most frequently tested SNP with respect to cancer risk. The risk of BrC among carriers of the ^241^Met-containg genotypes was found to be increased compared to wild-type carriers by some 6–10% under various genetic models [29–31]. Nevertheless, recent smaller studies conducted in Polish BrC population failed to provide evidence on any unambiguous effect with respect to BrC risk [32,33]. Other studies proposed the ^17893^G allele (rs1799796; intron 7; c.562-14A>G; ^17893^A/G) as providing protective effect against BrC (risk reduction of some 10%) [31].

Missense mutations in Rad51C were found to associate with hereditary breast and ovarian cancer (HBOC), which has been further confirmed in several subsequent studies on unselected ovarian cancer (OC). Nevertheless, there is still a considerable amount of studies which failed to find any association between Rad51C mutations and HBOC, what has usually been explained by very rare occurrence of these mutations [21,34–38]. Interestingly, none of the above cited studies identified Rad51C mutations associated with the BrC-only families.

The above cited reports prompted us to further contribute to our previous report on associations between genetic variability of Rad51C and the risk of BrC [39] by evaluating the associations between genetic variability of two more enzymes belonging to the Rad51 family—Rad51 and Xrcc3—and the risk of BrC. To this end, a total of 14 common SNPs in Rad51 and Xrcc3 (seven per *each* gene) were genotyped and tested for significant differences in their distributions between female BrC cases and controls. In addition to conventional analyses (single-site, SNP combinations and haplotype analyses), machine learning (ML) techniques (random forest and multifactor dimensionality reduction) providing increased statistical power and novel approach to cancer association studies [40] were used to explore main and nonlinear (epistatic) interactional effects of SNPs with respect to their association with BrC. In ML analyses, hereby described Rad51 and Xrcc3 genotypic data, supplemented with our previously reported genotypic data obtained for Rad51C [39], were used to broaden the picture of involvement of the Rad51 family in BrC.

## Material and methods

### Subjects

In this study, previously described groups of breast cancer patients and control subjects were used [39]. Briefly, the study group consisted of 132 female breast cancer patients of European descent aged 36–86 years (median age at the time of diagnosis of 57 years; interquartile range (IQR): 15 years) hospitalized at the Department of Oncology, Memorial Copernicus Hospital in Lodz, Poland in years 2007 and 2008 with histopathologically confirmed diagnosis of BrC. Only female patients with primary breast cancer tumor without metastases and without any history of previous anti-cancer treatment, undergoing curative resection therapy or chemotherapy, were enrolled. The control group consisted of 189 healthy cancer-free volunteer females of European descent, aged 35–54 years (median age at the time of examination of 43 years; IQR: 6 years), willing to undergo examinations. All subjects enrolled in the study were residents of Lodz district in central Poland.

Additional information on tobacco-smoking habits was collected for both controls and BrC cases and the individuals were classified as either never- or ever-smoker according to the criterion suggested by Pomerleau et al. [41]. According to this criterion, only those subjects who have smoked less than 19 cigarettes (a pack) during their lifetime, were classified as never-smokers, while the others were considered as ever-smokers. No data concerning the alcohol consumption was available for either controls or cases.

Written and informed consent for participation in this study was obtained from each subject enrolled prior to any experiments. The study was performed under the guidelines of the Helsinki Declaration for human research and was approved by the Bioethics Committee in the Nofer Institute of Occupational Medicine (resolution no. 5/2007). Characteristics of the breast cancer group and the control group are summarized in Table 1.

**Table 1:** Characteristics of the groups of subjects involved in the study.

Feature	BrC cases	Control subjects
Total number	132	189
Age [years]	57 [36–86] ^a^	43 [35–54]
Smoking status [never/ever]	55/63 (0.47/0.53) ^b^	120/68 (0.64/0.36)
Pack-years	11.3 [6.0–20.0] ^a^	2.0 [1.0–4.6]
Tumor staging ^c^		
T [1/2/3/4/x]	44/45/1/6/1	-
N [0/1/2/3/x]	44/24/10/2/20	-
Tumor grade		
G [1/2/3/x]	7/37/40/13	-

Numerical data for age and pack-years presented as median [range]. Smoking status [never/ever-smokers] expressed as absolute [relative] counts.

^a^ p << 0.001; BrC cases vs. controls; Mann-Whitney *U* test

^b^ p < 0.005; BrC cases vs. controls; two-sided Mid-P test

^c^ Tumor staging classification (absolute counts): T—describes the size of the primary tumor and its invasiveness, N—describes the regional lymph nodes involved, x—data unavailable.

### DNA isolation

Peripheral blood leukocytes were used to isolate the genomic DNA using the QIAamp DNA Blood Mini Kit (Qiagen, Germany). Manufacturer’s instructions were followed. RNA contamination was removed by digestion with 1 mg/ml RNase A (Qiagen, Hilden, Germany). DNA quantification, its purity and protein content were assessed using an Eppendorf BioPhotometer (Eppendorf, Hamburg, Germany) instrument. All DNA samples were stored at -80°C until further processing.

### Genotyping

**Fig 1:**
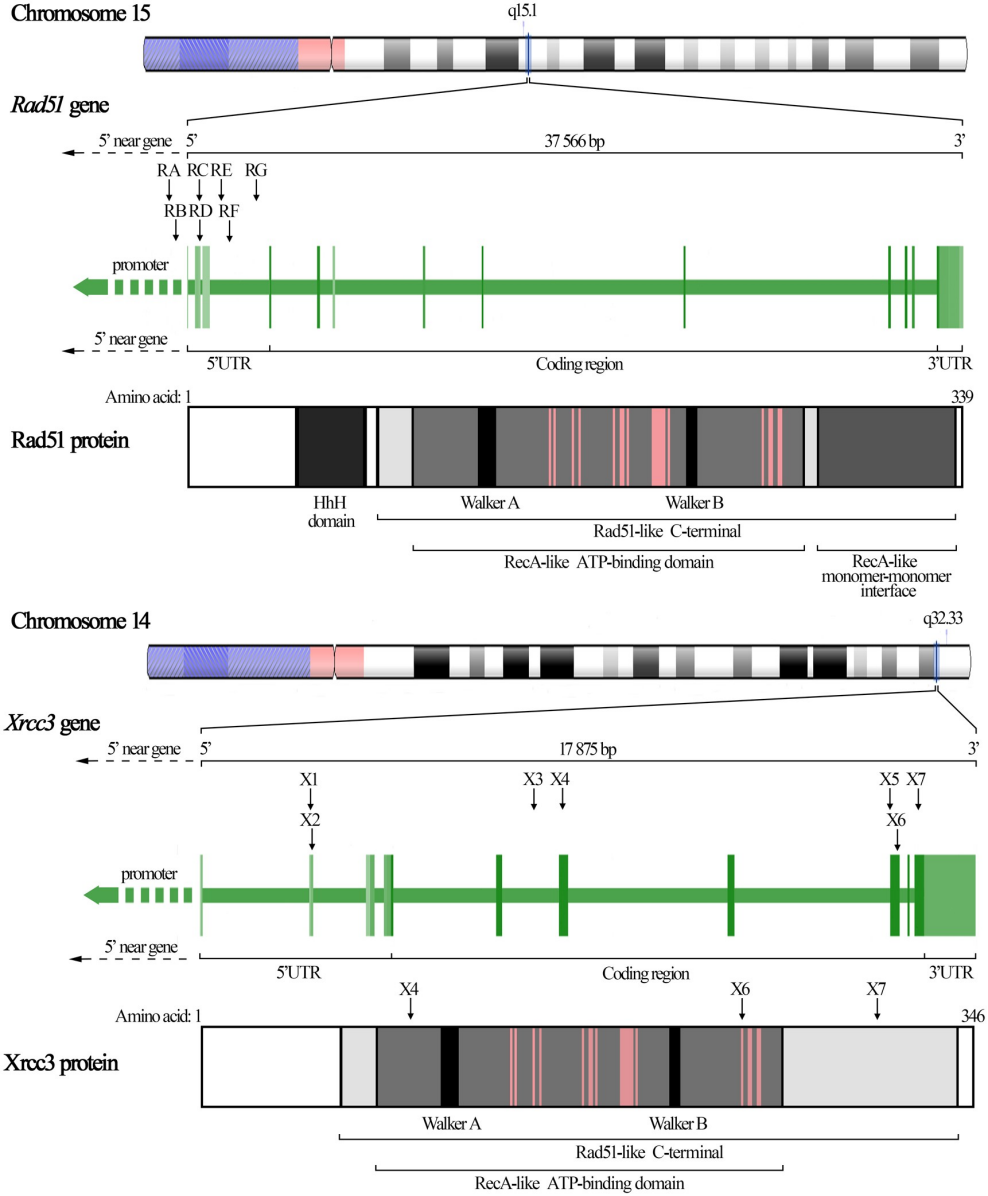
*Rad51* and *Xrcc3* gene and protein structures. *Rad51* and *Xrcc3* gene and protein structures are shown with graphic representation of the localization of all 14 SNPs investigated in this study. The positions of individual SNPs in respective genes are indicated by arrows annotated with respective working codes used in this study. Localizations of rs3212057 (X4), rs861539 (X6) and rs28903081 (X7) are indicated also within the Xrcc3 protein structure, as these were the only three SNPs localized within coding regions of exons. *Rad51* and *Xrcc3* are placed on chromosomes 15 and 14 and span 37 and 18 kb, respectively. Their protein products consist of 339 and 346 amino acids, respectively, showing high degree of conservativeness. They both share highly conserved Rad51-like C-terminal domain with approx. 250 amino acids (responsible for DNA binding) within which two subdomains can be distinguished: the RecA-like ATP-binding domain (responsible for ATP binding and hydrolysis) and the RecA-like monomer-monomer interface. The RecA-like ATP-binding domain embodies two highly conserved consensus motifs (Walker A and B) conferring ATP-binding and -hydrolysis activities. Distributed within this domain is also the multimere BRC interface (pink lines in the protein structure) facilitating interaction with DNA. X4, X6 and X7 are all localized within Rad51-like C-terminal domain, while the rs861539 (X6) hits exactly the threonine-241 taking part in interaction with DNA. Rad51 consists additionally of the helicase-hairpin-helicase (HhH) domain responsible for nonspecific DNA binding [42,45].

*Rad51* paralogs show a relatively high degree of conservativeness (for gene and protein conservativeness schemes see Fig 1 and S1 Fig) with missense changes being very rare within these genes, therefore, focus of this study was put on SNPs occurring predominantly in non-coding regions which may plausibly be involved in regulation of gene expression. A total of 14 SNPs with seven of them being localized in *Rad51* and additional seven in *Xrcc3* were analyzed. In the case of *Rad51*, SNPs occurring in the promoter, 5′UTR and intron 3 with the minor allele frequency (MAF) in the Caucasian population exceeding 10% (according to the dbSNP database [42]) were selected. In the case of *Xrcc3*, in addition to frequently analyzed rs1799794 (^4541^A/G), rs1799796 (^17893^A/G) and rs861539 (p.Thr241Met), SNPs localized in 5’UTR, intron 5, together with two missense SNPs in exons 6 and 10, for which the minor allele frequency in the Caucasian population was higher than 10%, were selected. Detailed information on SNPs analyzed in this study are provided in Table 2, while additional graphic representation of localization of all analyzed SNPs is provided in Fig 1. Plausible effects of selected SNPs in non-coding regions, as predicted by the PERFECTOS-APE *in-silico* method for prediction of regulatory functional effect of noncoding SNPs [43], are provided in Table 3. Prediction of classification of non-synonymous SNPs analyzed in the study, obtained by Polyphen-2 [44], can also be found in Table 3.

**Table 2:** Resume of SNPs genotyped in the study.

Working code	rs-code	Designation	Other designations	SNP position	MAF
*Rad51*					
RA	rs2619679	c.-1389T>A	^-4719^A/T	5' near gene (promoter)	T: 0.49
RB	rs5030789	c.-1271A>G	^-4601^A/G	5' near gene (promoter)	A: 0.42
RC	rs1801320	c.−98G>C	^135^G/C	Exon 2 (5'UTR)	C: 0.08
RD	rs1801321	c.−61G>T	^172^G/T	Exon 2 (5'UTR)	T: 0.42
RE	rs2619680	c.−3+795C>A	^1037^A/C	Intron 3	A: 0.50
RF	rs2619681	c.−3+1398T>C	^1640^C/T	Intron 3	T: 0.15
RG	rs2928140	c.−2−602G>C	^2972^C/G	Intron 3	G: 0.49
*Xrcc3*					
X1	rs1799794	c.−316A>G	^4541^A/G	Exon 2 (5'UTR)	G: 0.22
X2	rs45603942	c.−281C>T	^4576^C/T	Exon 2 (5'UTR)	*unknown*
X3	rs861530	c.194−571A>G	^9685^A/G	Intron 5	A: 0.31
X4	rs3212057	c.281G>A	^10343^G/A; p.Arg94His	Exon 6	A: 0.03
X5	rs1799796	c.562-14A>G	^17893^A/G	Intron 7 (IVS6'14)	G: 0.30
X6	rs861539	c.722C>T	^18067^C/T; p.Thr241Met	Exon 8	T: 0.39
X7	rs28903081	c.905G>A	p.Arg302His	Exon 10	*unknown*

SNP nomenclature, assigned working symbols, localization and minor allele frequencies (MAF) are provided for general European population according to data obtained from the dbSNP database [42]. Table partially taken from [46], nevertheless some corrections have been introduced due to data actualization in dbSNP database.

**Table 3:** Predicted effects of SNPs analyzed in the study.

#rs	Fold-change in transcription factor binding affinity caused by given SNP																				Polyphen-2	
	Code	Bmal-1	Clock	Creb-3	E2F-2	E2F-4	AP-1	jun-D	Myc	N-myc	NF-κB1	NRF1	p53	p63	Pax-6	Pax-8	RAR-α	RAR-γ	Sp1	Wt1	class	score
*Rad51*																						
rs2619679	RA	-	-	0.33	-	3.27	4.68	4.45	-	-	4.41	-	-	-	3.37	3.47	-	-	-	-	-	-
rs5030789	RB	-	0.18	-	0.21	0.08	-	-	0.24	0.22	-	3.57	9.02	3.95	5.38	-	3.72	-	-	-	-	-
rs1801320	RC	9.86	3.95	-	-	0.32	-	-	15.91	21.22	-	-	0.26	-	-	-	-	0.30	0.11	0.26	-	-
rs1801321	RD	-	-	0.11	0.05	6.24	-	-	0.16	-	7.32	0.10	-	-	3.61	22.98	0.32	3.72	0.09	21.65	-	-
rs2619680	RE	-	0.27	-	-	-	-	-	4.58	-	0.10	11.45	-	0.21	0.13	-	4.28	6.24	-	-	-	-
rs2619681	RF	0.07	-	-	-	-	0.16	0.09	0.17	0.29	0.24	-	-	-	-	-	-	-	-	-	-	-
rs2928140	RG	-	-	-	-	-	0.24	0.27	-	-	0.25	-	-	-	-	-	-	3.87	0.26	0.22	-	-
*Xrcc3*																						
rs1799794	X1	-	-	0.17	-	-	-	-	0.30	-	3.61	-	-	-	-	3.61	-	3.17	9.02	-	-	-
rs45603942	X2	-	-	5.59	-	-	0.29	-	-	-	-	-	-	-	-	-	3.50	14.68	-	-	-	-
rs861530	X3	0.04	0.29	-	0.26	3.02	-	-	0.20	0.09	-	-	8.93	5.65	-	-	-	-	-	-	-	-
rs3212057	X4	-	-	-	-	-	-	-	-	-	-	-	-	-	-	-	-	-	-	-	neutral	0.334
rs1799796	X5	0.17	0.13	6.00	-	-	-	-	-	-	-	-	-	-	-	5.43	-	0.21	-	-	-	-
rs861539	X6	-	-	-	-	-	-	-	-	-	-	-	-	-	-	-	-	-	-	-	deleterious	0.541
rs28903081	X7	-	-	-	-	-	-	-	-	-	-	-	-	-	-	-	-	-	-	-	neutral	0.001

Results of PERFECTOS-APE and Polyphen-2 *in-silico* analyses for non-coding and non-synonymous SNPs, respectively, analyzed in the study. Effects of non-coding SNP were predicted for several well-known transcription factors (TF) against the HOCOMOCO-11 [69] database and are provided as fold changes in respective TF binding affinity caused by given SNP, calculated as the ratio of probability of finding a better scoring TF binding motif by chance given the wild-type and variant SNP allele, respectively [43]. Only 3-fold or larger changes are shown, values above 3.0 indicate TF binding affinity upregulation, values below 0.33 indicate downregulation. All provided values are statistically significant at the level of p<0.05 or higher. For each non-synonymous SNP the classification of SNP provided by the Polyphen-2 algorithm together with respective probability value is provided [44].

All *Xrcc3* and all but one (rs1801321) *Rad51* SNPs were genotyped using the PCR-restriction fragment length polymorphism (PCR-RFLP) technique on a BioRad’s PTC-200 DNA Engine thermal cycler (BioRad, Hercules, CA, USA) instrument utilizing the Qiagen’s HotStarTaq PCR kit (Qiagen, Hilden, Germany). Used primer sequences, together with their basic characteristics and used PCR conditions, have already been provided in full details elsewhere [46], but are listed again for convenience in Table 4.

**Table 4:** Summary on primer sequences, amplicons, PCR cycling conditions and enzymatic digestion for individual SNPs genotyped in the study [46].

rs# of SNP	Primer sequences (Forward/Reverse)	Amplicon length	PCR ^a^	Enzymatic digestion ^b^
*Rad51*				
rs2619679 ^c^	5'-CCGTGCAGGCCTTATATGAT-3'	402 bp	95°C(45s) 60°C(45s) 72°C(60s) 28x	*HinfI (37°C/65°C)*
rs5030789 ^c^	5'-AGATAAACCTGGCCAACGTG-3'			*Hin1II (37°C/65°C)*
rs1801320	5'-AGACCGAGCCCTAAGGAGAG-3'	399 bp		*Mva*I (37°C/20 mM EDTA)
	5'-GAGGTCCACTTGTGTTTTCG-3'			
rs1801321 ^d^	TaqMan Pre-Designed SNP Genotyping Assay (Life Technologies; Assay ID: C___7482700_10)	*unavailable*	95°C(15s) 61.5°C(60s) 50x	*not applicable*
rs2619680	5'-ATTACAGGCCCCCACCAC-3'	485 bp	95°C(45s) 65°C(45s) 72°C(60s) 29x	*Hin6I (37°C/65°C)*
	5'-CCAGGCTAGATCCTCCCTTC-3'			
rs2619681	5'-ACATGCTTGCCAACACGATA-3'	245 bp	95°C(45s) 63°C(45s) 72°C(60s) 28x	*Alw26I (37°C/65°C)*
	5'-CATAACTGAGGGCTGATAACCA-3'			
rs2928140	5'-CGCTTCTGGCTATTTTGAAGT-3'	332 bp		*Eam1104I (37°C/65°C)*
	5'-TGAGGCAGGTAAATGGCTTC-3'			
*Xrcc3*				
rs1799794	5'-CACACTGCGGTCTTGCAG-3'	505 bp	95°C(45s) 64°C(45s) 72°C(60s) 28x	*BseGI (55°C/80°C)*
	5'-GGCTGGGTCTGGATACAAAA-3'			
rs45603942	5'-GGGATGCAGGTTCAACTGAC-3'	515 bp		*AluI (37°C/65°C)*
	5'-ATGAACCTCGCACCTGGTAG-3'			
rs861530	5'-CTGCAGGTGGCTCAGTGG-3'	497 bp		*Hin1II (37°C/65°C)*
	5'-CTCCCTAACAGCCTCCATGT-3'			
rs3212057	5'-CTTGCTCACCCCCATGAC-3'	467 bp		*Cfr42I (37°C/65°C)*
	5'-AATGGTAGGAACAGCGCAAG-3'			
rs1799796	5'-CAGAGTATGGGCACTGTGAGC-3'	400 bp		*AluI (37°C/65°C)*
	5'-CCGCATCCTGGCTAAAAATA-3'			
rs861539	5'-AAGAAGGTCCCCGTACTGCT-3'	452 bp		*Hin1II (37°C/65°C)*
	5'-CAGAGGTGCACACACCACAT-3'			
rs28903081	5-'CCTGCTTCCTGTTTCTCAGG-3'	445 bp		*Bsh1236I (37°C/65°C)*
	5'-AGGGAGAGGCAGAACATCC-3'			

^a^ PCR conditions are provided as temperature (duration) of denaturation, annealing and extension phase, respectively, followed by the number of cycles for a given SNP. Each PCR reaction was finished by a final 10 min-long extension phase.

^b^ Digestion/enzyme deactivation temperatures, respectively, are provided for each enzyme. Digestion was always performed for 20 minutes. In the case of *Mva*I, the reaction was stopped by adding 20 mM of EDTA according to the manufacturer’s instruction.

^c^ rs2619679 and rs5020789 were co-amplified in one PCR reaction tube using only one pair of primers and following the PCR reaction were divided into separate tubes and subjected to endonuclease digestion separately.

^d^ rs1801321 in Rad51 was genotyped by means of the real-time PCR technique using commercially available TaqMan SNP Genotyping Assay Kit and detailed conditions of the reaction are provided elsewhere [46].

Rs1801321 in *Rad51* was genotyped by the real-time PCR technique using the predesigned commercially available TaqMan SNP Genotyping Assay kit (Life Technologies, Carlsbad, CA, USA) and detailed conditions of the reaction have also been previously provided [46].

In the case of *Rad51C*, no SNPs within this gene were genotyped in this study. Instead, genotypic data of a set of eight SNPs previously described in our recent study on breast cancer [39] were used. These SNPs included rs302874 (hereby designated RcA), rs12946522 (RcB), rs302873 (RcC), rs16943176 (RcD), rs12946397 (RcE), rs28910276 (RcF), rs17222691 (RcG) and rs28363302 (RcH). More details on genotyping and the results of analysis of associations between these SNPs and BrC can be find elsewhere [39].

### Statistical analyses

#### Single-locus analyses

For all SNPs, both absolute and relative genotypic frequencies are provided. The Hardy-Weinberg equilibrium (HWE) in controls was tested by a goodness-of-fit chi-square test. For each investigated SNP, possible associations with BrC risk at both allelic and genotypic level were sought for by Fisher’s exact test (allelic level) or unconditional logistic regression (genotypic level) in a series of separate univariate (i.e. single-site) analyses. Associations with BrC are expressed as either raw (allelic level) or age-and-smoking-status-adjusted (genotypic level) odds ratios (ORs) with corresponding 95% confidence intervals (95% CI). Dominant, recessive, and additive genetic model, together with direct comparison of *variant* versus *wild-type* homozygotes were assumed. Significance was inferred for p<0.05.

#### Analysis of Rad51/Xrcc3 SNP combinations

*Rad51* and *Xrcc3* SNP with statistically significant outcomes from single-site analyses were further involved in analysis of association between their mutual combinations and BrC. BrC risk associated with combinations of the so-called high-risk genotypes in these polymorphic sites was estimated by means of age-and-smoking-status-adjusted unconditional logistic regression and expressed as ORs with corresponding 95% CIs.

#### Analysis of Rad51 and Xrcc3 haplotypes

Linkage disequilibrium (LD) and haplotype reconstruction were performed by means of an expectation-maximization algorithm implemented in the Haploview package [47]. Briefly, “strong LD” blocks were recognized based on normalized measure of allelic association |*D’*| [48] according to the confidence interval method proposed by Gabriel et al. [49]. As earlier described [39], haplotypes reconstructed within each “strong LD” block were tested for differences in their frequencies between the control and cancer group and the significance of such differences was assessed using a two-sided exact mid-P test. Possible linkage of haplotypes with BrC was expressed by means of OR with corresponding 95% CI and significance was inferred for p<0.05.

### Analyses based on machine learning techniques

In these analyses, SNPs for which no variability was found in single-locus analyses (rs45603942 (X2) and rs28903081 (X7) in *Xrcc3*; rs28910276 (RcF) in *Rad51C* [39]) were omitted and subjects with any lacks in data were excluded. Therefore, a total of 19 SNPs (7 SNPs in *Rad51*, 5 SNPs in *Xrcc3* and 7 SNPs in *Rad51C*) were involved.

#### RF-based analysis of associations between predictors and BrC

In addition to above described single-locus analyses, simple associations between BrC and analyzed SNPs were assessed by means of the random forest (RF) machine learning strategy. The Breiman-Cutler permutation variable importance (VIMP) [50] was used to measure and rank the strength of such associations. In our study, we used the *randomForestSRC* package for R obtained from the CRAN repository [51], using which we employed a robust strategy allowing us to reliably validate and statistically infer on the ranking of all analyzed SNPs with respect to their ability to accurately predict the BrC/control status. Detailed description of this strategy is provided in Part A of the S1 File.

The whole procedure was performed twice. In the first run, only SNPs were considered as possible predictors of the BrC case/control status, which provided us with “raw” results, while the second one considered SNPs together with subjects’ age (dichotomized with respect to median age) and smoking status (never/ever smoker), providing the VIMP-based ranking of SNPs allowed for interactions with these two common confounders. Levels of significance were obtained by permutation testing (see the Part A in S1 File for further details) and statistical significance was inferred for p<0.05.

#### Random forest analysis of epistatic interactions

To distinguish between main and interactive (i.e. epistatic) effects of SNPs on BrC, direct analysis of pure epistatic interactions and their association with BrC was performed using a permutation-based machine learning strategy relying on RF methodology termed the permuted random forest (pRF). pRF detects and quantifies pure interaction between selected SNPs and estimates how much it contributes to the model prediction power. We have implemented this method in R using the *randomForestSRC* [51] and *permutations* [52] packages obtained from the CRAN repository according to a thorough description of algorithm provided by Li et al. [53] with minor modifications allowing us to perform analysis of interactions between pairs as well as among triplets of SNPs (i.e. 2-way and 3-way interactions). Subjects’ age (dichotomized with respect to age median) and smoking status (never/ever smoker) were also involved in the analysis as possible confounders. The same RF model as the one used to obtain the VIMP-based ranking of simple associations between SNPs and BrC was used in this analysis. All possible 2-way and 3-way combinations of predictors (SNPs and confounders) were analyzed and the strength of associations between individual combinations of predictors and BrC was measured by the so-called differential error (ΔE; see the Part B in the S1 File). The predictor combination with the highest value of ΔE was considered as the best one being in the strongest association with BrC. Detailed description of the algorithm used can be found in Part B of the S1 File.

#### Analysis of epistatic interactions using dimensionality reduction approach

As an alternative approach for elucidating the epistatic interactions among predictors (SNPs and subject’s age and smoking status as confounders) and their associations with BrC, the model-based multifactor dimensionality reduction (MB-MDR) was used.

The algorithm was implemented using the *mbmdr* package for R obtained from the CRAN repository [54] and is described in more details in Part C of the S1 File. MB-MDR uses a constructive induction technique to merge multi-locus genotypes into a one-dimensional construct, assigning each analyzed combination of genotypes to either “high-risk”, “low-risk”, or a “no-evidence” (or “non-informative”) category. Such new predictive variable with three states (H, L, 0) was then tested for association with the risk of BrC and such association then expressed by means of Wald statistic, OR and respective *p* value (separately for the “high-risk” and “low-risk” category, where appropriate). Only 2-way and 3-way interactions were analyzed, and permutation testing was used to correct the obtained *p*-levels for multiple hypotheses testing.

## Results

### Single-locus analyses of associations between *Rad51* and *Xrcc3* SNPs and breast cancer

Single site analyses of 14 SNPs within *Rad51* and *Xrcc3* in terms of their possible associations with BrC were performed. All relevant results including the counts (frequencies) in the BrC and control groups together with logarithmic regression-derived ORs adjusted to age and smoking status are presented in Tables 5A and 6A. For all investigated SNPs, observed genotype frequencies in control groups were in agreement with those predicted by the Hardy-Weinberg law.

**Table 5:** Observed genotype and haplotype frequencies together with respective odds ratios (ORs) and corresponding 95% confidence intervals for *Rad51* polymorphisms in breast cancer cases and control subjects.

**A**	**SNP**	**BrC**	**Control**	**OR [95% CI]** ^a^^,^^b^	
	**(RA)** rs2619679				
	AA	32 (0.250)	49 (0.265)	A: 1.0 [0.7–1.4]	
	AT	73 (0.570)	98 (0.530)	D: 1.0 [0.6–1.7]	All: 1.0 [0.7–1.4]
	TT	23 (0.180)	38 (0.205)	R: 1.0 [0.5–1.7]	W/R: 0.9 [0.5–1.9]
	**(RB)** rs5030789				
	GG	41 (0.320)	51 (0.277)	A: 0.7 [0.5–1.0] ^e^	
	GA	71 (0.555)	90 (0.489)	D: 0.7 [0.4–1.3]	All: 0.7 [0.5–1.0] ^f^
	AA	16 (0.125)	43 (0.234)	**R: 0.5 [0.3–1.0]** ^d^	**W/R: 0.5 [0.2–1.0]** ^d^
	**(RC)** rs1801320				
	GG	93 (0.830)	109 (0.845)	A: 1.5 [0.9–2.6]	
	GC	11 (0.098)	19 (0.147)	D: 1.2 [0.6–2.5]	All: 1.6 [0.9–2.8]
	CC	8 (0.071)	1 (0.008)	**R: 10.6 [1.9–198]** ^d^	**W/R: 9.8 [1.8–184]** ^d^
	**(RD)** rs1801321				
	GG	43 (0.326)	72 (0.381)	A: 1.1 [0.8–1.6]	
	GT	72 (0.545)	89 (0.471)	D: 1.2 [0.7–2.0]	All: 1.1 [0.8–1.5]
	TT	17 (0.129)	28 (0.148)	R: 1.0 [0.5–2.0]	W/R: 1.2 [0.5–2.4]
	**(RE)** rs2619680				
	AA	30 (0.244)	49 (0.288)	A: 1.0 [0.7–1.5]	
	AC	71 (0.577)	89 (0.524)	D: 1.1 [0.6–1.9]	All: 1.1 [0.8–1.5]
	CC	22 (0.179)	32 (0.188)	R: 1.0 [0.5–1.8]	W/R: 1.0 [0.5–2.2]
	**(RF)** rs2619681				
	CC	100 (0.787)	143 (0.769)	A: 0.8 [0.5–1.3]	
	CT	27 (0.213)	40 (0.215)	D: 0.8 [0.5–1.5]	All: 0.8 [0.5–1.4]
	TT	0 (0.000)	3 (0.016)	R: 0.0 [*NA-NA*]	W/R: 0.0 [*NA-NA*]
	**(RG)** rs2928140				
	CC	28 (0.217)	49 (0.265)	A: 1.0 [0.7–1.5]	
	CG	77 (0.597)	97 (0.524)	D: 1.2 [0.7–2.2]	All: 1.1 [0.8–1.4]
	GG	24 (0.186)	39 (0.211)	R: 0.8 [0.5–1.5]	W/R: 1.0 [0.5–2.1]
**B**	**Haplotype (block 1)**	**BrC**	**Control**	**OR [95% CI]** ^c^	
	(RD,RE,RF): TAC	104 (0.405)	142 (0.377)	1.1 [0.9–1.3]	
	(RD,RE,RF): GCC	93 (0.362)	126 (0.336)	1.1 [0.8–1.6]	
	(RD,RE,RF): GAC	32 (0.124)	59 (0.156)	0.8 [0.5–1.2]	
	(RD,RE,RF): GCT	28 (0.108)	46 (0.122)	0.9 [0.5–1.4]	
	(RD,RE,RF): TCC	0 (0.000)	2 (0.006)	*NA*	
	(RD,RE,RF): GAT	0 (0.000)	1 (0.003)	*NA*	

**A)** Single-site analyses showed statistically significant BrC risk-reducing effect for rs5030789 and rs1801320 under recessive genetic model, only. **B)** Results of haplotype analysis for *Rad51* SNPs showed no observable associations between *Rad51* haplotypes and BrC. Data presented as absolute (relative) frequencies of individual genotypes observed in BrC cases and controls; *NA*—not applicable;

^a^ Genetic model employed in order to analyze the association between genotype distribution and cancer: A—additive genetic model; D—dominant genetic model; R—recessive genetic model; All—direct comparison of the frequency of alleles; W/R—comparison of wild-type and rare homozygous genotypes.

^b^ OR values adjusted for age and smoking status, together with 95% CI determined by logistic regression.

^c^ Significant differences in genotype distributions between the BrC and control groups were sought for by two-sided exact mid-P test.

^d^ p < 0.05.

^e^ p = 0.06.

^f^ p = 0.07.

**Table 6:** Observed genotype and haplotype frequencies together with respective odds ratios (ORs) and corresponding 95% confidence intervals for *Xrcc3* polymorphisms in breast cancer cases and control subjects.

**A**	**SNP**	**BrC**	**Control**	**OR [95% CI]**^a^^,^^b^	
	**(X1)** rs1799794				
	AA	68 (0.535)	100 (0.610)	Add: 1.1 [0.8–1.6]	
	AG	51 (0.402)	52 (0.317)	Dom: 1.2 [08–2.0]	All: 1.2 [0.8–1.7]
	GG	8 (0.063)	12 (0.073)	Rec: 0.9 [0.3–2.2]	W/R: 0.9 [0.3–2.5]
	**(X2)** rs45603942				
	CC	120 (1.000)	186 (1.000)	Add: *NA*	
	CT	0 (0.000)	0 (0.000)	Dom: *NA*	All: *NA*
	TT	0 (0.000)	0 (0.000)	Rec: *NA*	W/R: *NA*
	**(X3)** rs861530				
	GG	53 (0.438)	79 (0.434)	Add: 0.9 [0.6–1.4]	
	GA	59 (0.488)	88 (0.484)	Dom: 1.0 [0.6–1.6]	All: 1.0 [0.7–1.4]
	AA	9 (0.074)	15 (0.082)	Rec: 0.8 [0.3–1.9]	W/R: 0.8 [0.3–1.9]
	**(X4)** rs3212057				
	GG	125 (1.000)	173 (0.961)	Add: 0.0 [*NA-NA*]	
	GA	0 (0.000)	7 (0.039)	Dom: 0.0 [*NA-NA*]	**All: 0.0 [0.0-*NA*]**^d^
	AA	0 (0.000)	0 (0.000)	Rec: *NA*	W/R: *NA*
	**(X5)** rs1799796				
	AA	50 (0.417)	75 (0.412)	Add: 1.2 [0.8–1.6]	
	AG	48 (0.400)	84 (0.462)	Dom: 1.0 [0.6–1.7]	All: 1.1 [0.8–1.6]
	GG	22 (0.183)	23 (0.126)	Rec: 1.7 [0.9–3.4]	W/R: 1.6 [0.8–3.3]
	**(X6)** rs861539				
	CC	57 (0.449)	71 (0.436)	Add: 1.0 [0.7–1.5]	
	CT	56 (0.441)	74 (0.454)	Dom: 0.9 [0.6–1.5]	All: 1.0 [0.7–1.4]
	TT	14 (0.110)	18 (0.110)	Rec: 1.3 [0.6–2.8]	W/R: 1.4 [0.6–3.2]
	**(X7)** rs28903081				
	GG	121 (1.000)	188 (1.000)	Add: *NA*	
	GA	0 (0.000)	0 (0.000)	Dom: *NA*	All: *NA*
	AA	0 (0.000)	0 (0.000)	Rec: *NA*	W/R: *NA*
**B**	**Haplotype (block 1)**	**BrC**	**Control**	**OR [95% CI]**^c^	
	(X1,X3): AG	171 (0.684)	246 (0.669)	1.1 [0.8–1.5]	
	(X1,X3): GA	65 (0.259)	79 (0.215)	1.3 [0.9–1.9]	
	(X1,X3): AA	14 (0.056)	41 (0.110)	**0.5 [0.3–0.9]**^d^	
	(X1,X3): GG	0 (0.000)	2 (0.006)	*NA*	
	**Haplotype (block 2)**	**BrC**	**Control**	**OR [95% CI]**^c^	
	(X5,X6): GC	93 (0.370)	128 (0.348)	1.1 [0.8–1.5]	
	(X5,X6): AT	82 (0.327)	125 (0.341)	0.9 [0.7–1.3]	
	(X5,X6): AC	73 (0.294)	113 (0.309)	0.9 [0.6–1.3]	
	(X5,X6): GT	2 (0.009)	1 (0.002)	2.9 [0.3–32.6]	

**A)** Single-site analyses showed statistically significant BrC risk-reducing effect for variant rs3212057 (^10343^A) allele at allelic level, only. **B)** Results of haplotype analysis for *Rad51* SNPs revealed statistically significant BrC risk reducing effect for ^4541^A/^9685^A (X1/X3; rs1799794/rs861530) *Xrcc3* haplotype. Data presented as absolute (relative) frequencies of individual genotypes observed in BrC cases and controls; *NA*—not applicable.

^a^ Genetic model employed in order to analyze the association between genotype distribution and cancer: A—additive genetic model; D—dominant genetic model; R—recessive genetic model; All—direct comparison of the frequency of alleles; W/R—comparison of wild-type and rare homozygous genotypes.

^b^ OR values adjusted for age and smoking status, together with 95% CI determined by logistic regression.

^c^ Significant differences in genotype distributions between the BrC and control groups were sought for by two-sided exact mid-P test.

^d^ p < 0.05.

Out of the 7 *Rad51* SNPs analyzed in this study, only rs5030789 (^-4601^A/G; RB) and rs1801320 (^135^G/C; RC) showed differences in genotype frequencies between the BrC and control groups.

As long as the rs5030789 is concerned, the rare ^-4601^AA genotype was found to be almost twice less common among BrC cases compared to controls (12.5% vs. 23.4%), hence it was associated with significantly decreased BrC risk under recessive genetic model (OR = 0.5, 95%CI: 0.3–1.0; p<0.05) as well as in direct comparison between *wild-type* and *variant* homozygotes (OR = 0.5, 95% CI: 0.2–1.0; p<0.05). According to outcomes of the analysis under additive genetic model, each copy of the ^-4601^A allele was associated with approximately 30% reduction in odds of BrC, although this outcome remained just beyond the edge of statistical significance (OR = 0.7, 95% CI: 0.5–1.0; p = 0.06). It is, however, considerably similar to an outcome obtained from direct comparison of allelic frequencies, in which the ^-4601^A allele was also found to be less frequent among BrC cases compared to controls (40.2% vs. 47.8%) and thus rendering some 30% reduction in BrC risk (OR = 0.7, 95% CI: 0.5–1.0; p = 0.07), yet just beyond the edge of statistical significance, too. No statistically significant outcomes were obtained for this SNP under dominant genetic model (Table 5A).

Concerning the rs1801320, the rare ^135^CC genotype was found to be significantly more abundant among BrC cases (7.1% vs. 0.8%) hence can be assumed as associated with increased risk of BrC under recessive genetic model (OR = 10.6, 95% CI: 1.9–198; p<0.05) as well as in direct comparison between *wild-type* and *variant* homozygotes (OR = 9.8, 95% CI: 1.8–184; p<0.05). However, no statistically significant outcomes were found under dominant or additive genetic models as well as in direct comparison of allelic frequencies for this SNP (Table 5A).

In the case of *Xrcc3*, two out of seven analyzed SNPs (rs45603942 (^4576^C/T; X2) and rs28903081 (c.905G>A; X7)) did not show any genetic variability as only the *wild-type* homozygotes were observed in both the BrC and control groups. Moreover, for rs3212057 (^10343^G/A; X4) no *variant* homozygotes were observed in this study. Although the distribution of genotypes for this SNP was found to differ between BrC cases and controls, with heterozygotes being abundant only among controls compared to BrC cases (3.9% vs. 0.0%), logistic regression adjusted to age and smoking-status did not, however, render this difference as statistically significant under either dominant or additive genetic model with respective ORs being unavailable due to zero frequency of heterozygotes among BrC subjects. Recessive genetic model and direct comparison between *wild-type* and *variant* homozygotes were not possible for this SNP. The only statistically significant outcome for this SNP was thus observed at allelic level, where the ^10343^A allele was found to be associated with reduced risk of BrC (OR = 0.0, 95% CI unavailable; p<0.05; Table 6A). No statistically significant outcomes for any other *Xrcc3* SNPs were found under any of the models examined.

### Linkage disequilibrium and haplotype analysis

**Fig 2:**
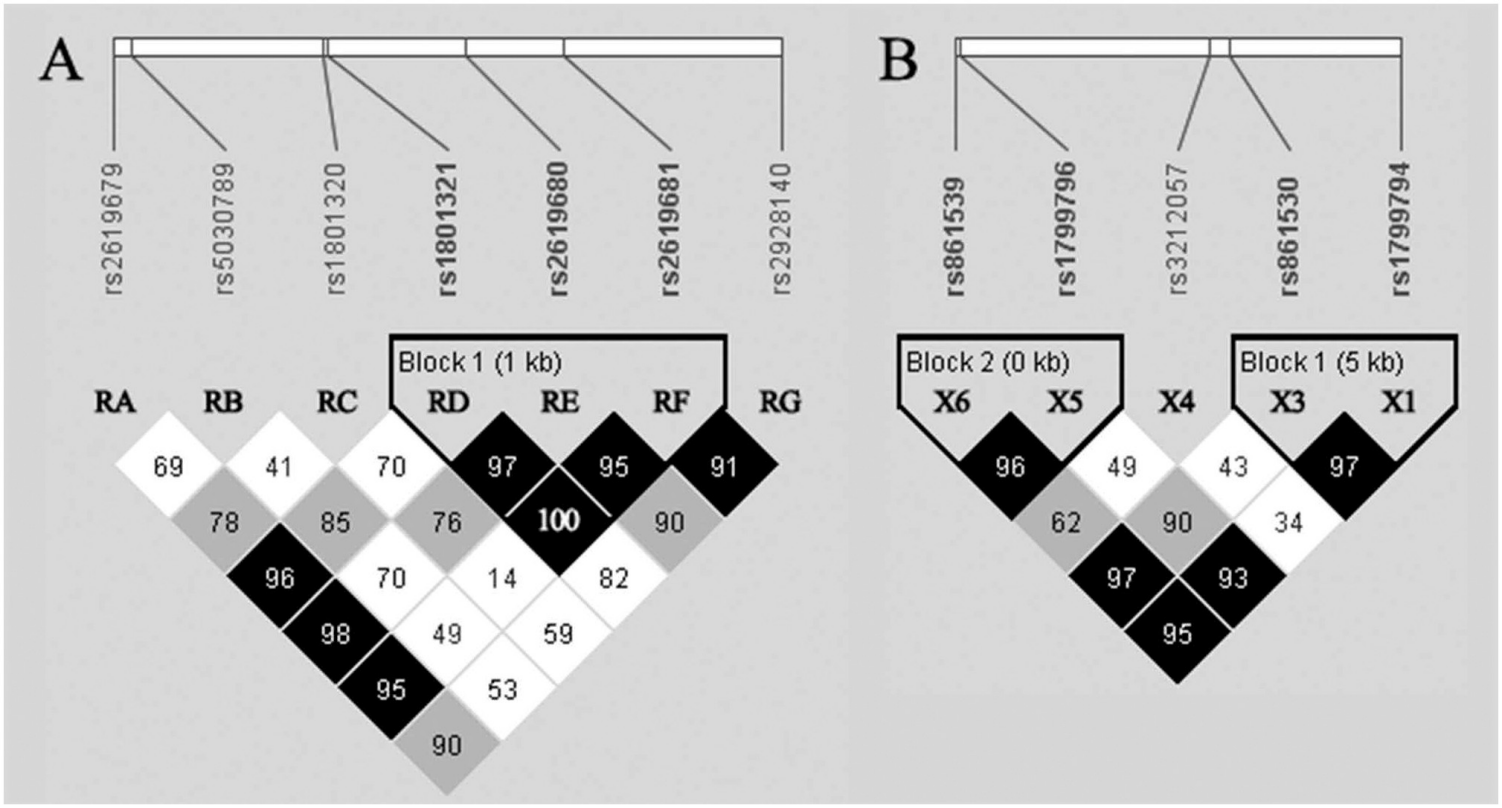
The map of LD among analyzed SNPs in (A) *Rad51* and (B) *Xrcc3*. In the case of *Xrcc3*, rs45603942 (X2) and rs28903081 (X7) are not shown as these SNPs were not considered in haplotype analysis due to lack of any genetic variability observed in these loci. For a given pair of SNP, the values in the map are the values of normalized measure of allelic association (|*D’*|, provided as percentages) and the color scheme represents the corresponding confidence bounds: dark gray—strong evidence of LD; light-gray—inconclusive; white—strong evidence of recombination [48]. Identified LD blocks are indicated by solid lines encompassing respective SNPs.

Analysis of non-random associations between the investigated *Rad51* SNPs revealed a 1-kb long block of “strong LD” spanning from rs1801321 (^172^G/T; RD) in 5’UTR of exon 1, through rs2619680 (^1037^A/C; RE) in intron 1, to rs2619681 (^1640^C/T; RF) in intron 1 (Fig 2A). Within this LD block, four common (^172^T/^1037^A/^1640^C; ^172^G/^1037^C/^1640^C; ^172^G/^1037^A/^1640^C; ^172^G/^1037^C/^1640^T) and two rare (^172^T/^1037^C/^1640^C, ^172^G/^1037^A/^1640^T) haplotypes were reconstructed. Common haplotypes encompassed together 99.4% of all subjects (Table 5B). Nevertheless, no significant associations with BrC risk were found for any of the haplotypes reconstructed.

In the case of *Xrcc3*, rs45603942 (^4576^C/T; X2) in 5’UTR of exon 1 and rs28903081 (c.905G>A; X7) in exon 7 were not included in the LD analysis due to lack of any observed variability in our study. The LD analysis revealed two blocks of “strong LD”, one spanning across 5 kb from rs1799794 (^4541^A/G; X1) in 5’UTR of exon 2 to rs861530 (^9685^A/G; X3) in intron 5, while the other one spanning over 174 bp from rs1799796 (^17893^A/G; X5) in intron 7 to rs861539 (^18067^C/T; X6) in exon 8 (Fig 2B).

Within the first LD block (X1-X3), three common (^4541^A/^9685^G, ^4541^G/^9685^A, ^4541^A/^9685^A) and one rare (^4541^G/^9685^G) haplotype were reconstructed, with the common haplotypes encompassing 99.7% of all subjects (Table 6B). Out of these haplotypes, only the ^4541^A/^9685^A common haplotype was found to be significantly associated with reduced risk of BrC, as it was significantly less abundant among BrC cases compared to controls, resulting in around 2-fold reduction of the odds of BrC comparing to carriers of all other haplotypes together (5.6% vs. 11.0% among BrC cases and controls, respectively; OR = 0.5; 95% CI: 0.3–0.9; p<0.05). Within the second (X5-X6) LD block, three common (^17893^G/^18067^C,^17893^A/^18067^T, ^17893^A/^18067^C) and one rare (^17893^G/^18067^T) haplotypes were reconstructed, with common haplotypes encompassing 99.5% of all subjects. Nevertheless, none of the haplotype reconstructed within this LD block was associated with the risk of BrC (Table 6B).

### Associations between *Rad51*/*Xrcc3* SNP combinations and BrC

Only those SNPs for which statistically significant outcomes in single-site analyses were revealed were involved in this analysis (i.e. rs5030789 (^-4601^A/G; RB), rs1801320 (^135^G/C; RC) and rs3212057 (^10343^G/A; X4)). Combinations of respective high-risk genotypes (i.e. rs5030789 (RB) ^-4601^G/G or ^-4601^G/A, rs1801320 (RC) ^135^C/C and rs3212057 (X4) ^10343^G/G) were tested for association with BrC against respective genotype combinations encompassing the lowest possible number of high-risk genotypes (which was 0 in the case of RB/RC, RC/X4 combinations and 1 in the case of RB/X4, RB/RC/X4 combinations, as for these combinations no subjects with low-risk-only genotype combinations were found). Obtained results are summarized in Table 7.

**Table 7:** Associations of *Rad51*/*Xrcc3* SNP combinations with breast cancer.

*Rad51*		*Xrcc3*	Rates (high-risk/low-risk) among cases vs. controls	OR [95% CI]
(RB) rs5030789 (^-4601^GG/^-4601^GA)	(RC) rs1801320 (^135^CC)	(X4) rs3212057 (^10343^GG)		
x	x		8/16 vs. 0/36	7.3 [2.1–25.8] ^b^
x		x	109/16 vs. 130/46 ^a^	2.2 [1.2–4.3] ^c^
	x	x	7/0 vs. 1/6	184.9 [3.1–11172.8] ^c^
x	x	x	7/16 vs. 0/38^a^	8.1 [2.3–28.9] ^b^

Only those SNPs for which significant associations with BrC risk were found in previous single-site analyses were included. Data presented as number of cases vs. controls carrying the combination of high-risk/low-risk genotypes in cross-marked SNP loci. Respective high-risk genotypes are provided in the header of the table.

^a^ Subjects carrying the genotype combinations containing 1 high-risk genotype used as reference group.

^b^ p < 0.005

^c^ p < 0.05

Significant associations with BrC risk were found for all three possible two-way combinations (i.e. rs5030789 (RB) ^-4601^G/G or ^-4601^G/A & rs1801320 (RC) ^135^C/C, p<0.002; rs5030789 (RB) ^-4601^G/G or ^-4601^G/A & rs3212057 (X4) ^10343^G/G, p<0.02; rs1801320 (RC) ^135^C/C & rs3212057 (X4) ^10343^G/G, p<0.02) as well as for the three-way combination (rs5030789 (RB) ^-4601^G/G or ^-4601^G/A & rs1801320 (RC) ^135^C/C & rs3212057 (X4) ^10343^G/G, p<0.002) of high-risk genotypes listed above, with generally higher levels of significance compared to *p* values obtained when individual SNPs were tested in single-site analyses. Carriers of these combinations of high-risk genotypes were at increased risk of BrC comparing to those carrying the genotype combinations with maximum possible number of low-risk genotypes (RB&RC: OR = 7.3; 95% CI: 2.1–25.8; RB&X4: OR = 2.2; 95% CI: 1.2–4.3; RC&X4: OR = 184.9; 95% CI: 3.1–11172.8; RB&RC&X4: OR = 8.1; 95% CI: 2.3–28.9). Detailed case/control rates are provided in Table 7.

### RF-based analysis of associations between analyzed SNPs and BrC

As for rs45603942 (X2) and rs28903081 (X7) in *Xrcc3* as well as for rs28910276 (RcF) in *Rad51C* no variability was found in single-locus analyses, 7 SNPs in *Rad51*, 5 SNPs in *Xrcc3* and 7 SNPs in *Rad51C* were further included in RF-based analysis of the strength of their associations with BrC. Intermediate outcomes based on which the best RF-based models were selected are provided in the S2 Table. Performance characteristics of these RFs are provided in S3 Table.

**Fig 3:**
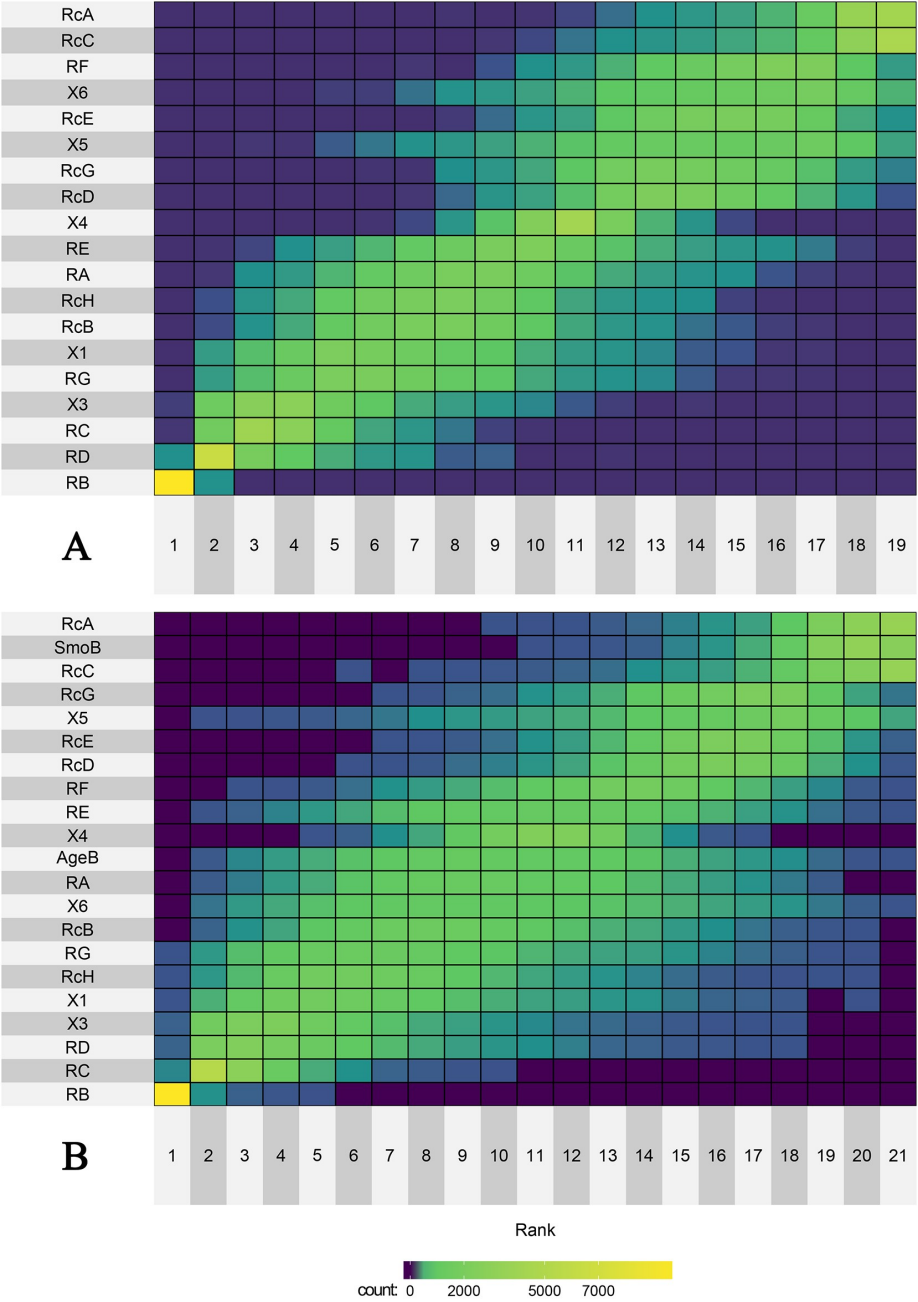
Results of bootstrap analysis of the VIMP-based ranking of predictors. Bootstrap estimate of distribution of the VIMP-based ranks of analyzed predictors obtained either **(A)** without or (**B)** with subjects’ age (AgeB) and smoking status (SmoB) as covariates. Individual SNPs and/or covariates are ordered according to their weighted average ranks as revealed by the resampling with replacement procedure inherent to the RF methodology following 10,000 runs. Color of each rectangle indicate the total number of bootstrap samples in which a given rank in the VIMP-based ranking was recorded for respective predictor. The more yellowish the color, the higher the total number of samples with respective rank. (Numerical data are provided in S3 Table).

When only SNPs were tested for associations with BrC (i.e. without covariates), obtained VIMP values (Table 8) suggest rs5030789 (RB), rs1801321 (RD), rs1801320 (RC), rs861530 (X3), and rs2928140 (RG) as the five best BrC/control predictors with the strongest association with BrC/control status. Moreover, the VIMPs of rs5030789 (RB), rs1801321 (RD), rs1801320 (RC) and rs3212057 (X4) were all found to be statistically significant (p<0.005 for RB; p<0.05 for RD, RC, and X4), while the VIMP value of rs861530 (X3) remained close to the edge of statistical significance (p = 0.074). The results of bootstrapping the RF-based ranking procedure are shown in Fig 3A. It is of note that the rs5030789 (RB) SNP was ranked 1^st^ in the vast majority of all bootstrapped RF models (99.6% of all 10,000 RFs; Part A in S4 Table) and based on derived weighted average ranks it seems to be confirmed as the best predictor with the strongest association with the BrC/control status. Besides that, six predictors with the lowest weighted average ranks in Fig 3A (i.e. the six best ones) were the same as in the ranking obtained based on observed VIMP values (rs5030789 (RB), rs1801321 (RD), rs1801320 (RC), rs861530 (X3), rs2928140 (RG), and rs1799794 (X1)).

**Table 8:** Ranking of all analyzed predictors (SNPs, covariates) according to their VIMP-based association with BrC.

Predictor	w/out covariates			with covariates		
	VIMP	p_VIMP_	Rank	VIMP	p_VIMP_	Rank
RB	0.0181	**0.0048**	1	0.0108	**0.0165**	1
RD	0.0093	**0.0370**	2	0.0070	**0.0416**	2
RC	0.0075	**0.0432**	3	0.0063	**0.0355**	4
X3	0.0071	0.0742	4	0.0054	0.0859	5
RG	0.0059	0.1068	5	0.0067	0.0603	3
X1	0.0052	0.1310	6	0.0041	0.1237	7
RcH	0.0038	0.1282	7	0.0019	0.1660	11
RA	0.0035	0.1428	8	0.0021	0.1981	10
RE	0.0029	0.1671	9	0.0003	0.3995	14
RcB	0.0029	0.1418	10	0.0036	0.0890	8
X4	0.0017	**0.0268**	11	0.0010	0.0720	12
RcE	0.0009	0.3006	12	-0.0020	0.8351	18
RcD	0.0007	0.3196	13	-0.0002	0.4791	16
X5	0.0002	0.4097	14	-0.0018	0.6128	17
X6	-0.0001	0.4373	15	0.0045	0.1252	6
RF	-0.0004	0.4488	16	0.0007	0.3068	13
RcG	-0.0009	0.5739	17	0.0002	0.3839	15
RcC	-0.0042	0.8095	18	-0.0046	0.9041	21
RcA	-0.0050	0.8714	19	-0.0024	0.7049	19
Age	-	-	-	0.0031	0.1407	9
Smoking	-	-	-	-0.0042	0.9355	20

Table presents the variable importance (VIMP) value for each predictor, together with respective significance level (p_VIMP_) obtained by permutation test using 10,000 permutations and corresponding VIMP-based ranking, as obtained in RF-based analysis with/without age and smoking status as covariates. The higher the VIMP value the stronger the association of a given predictor with BrC. P_VIMP_ values in bold indicate statistically significant association.

When subjects’ age and smoking status were added to analysis, obtained VIMP-based ranking (Table 8) indicated rs5030789 (RB) and rs1801321 (RD) again as the two best predictors with the strongest association with the BrC/control status, with rs1801320 (RC), rs861530 (X3), and rs2928140 (RG) running up on consecutive three places, with some minor shuffling compared to their ranks obtained in analysis not involving covariates, though. Again, VIMPs of rs5030789 (RB), rs1801321 (RD), rs1801320 (RC) were statistically significant (p<0.05 for all three SNPs), while those of rs2928140 (RG) and rs3212057 (X4) remained close to the edge of statistical significance (p = 0.060 for RG and p = 0.072 for X4). Fig 3B shows the results of bootstrapping the RF-based ranking procedure. Again, the rs5030789 (RB) SNP was ranked 1^st^ in the vast majority of all bootstrapped RF models (98.5% of all 10,000 RFs; Part B in S4 Table) and based on derived weighted average ranks it was confirmed as the best predictor with the strongest association with the BrC/control status. Predictors which were found among the top based on their observed VIMP values (such as rs1801321 (RD), rs2928140 (RG), rs1801320 (RC), and rs861530 (X3)) were again among the top predictors also based on their weighted average ranks.

### Analysis of epistatic interactions

The possible effects of epistatic interactions among SNPs on BrC/control status were analyzed by the pRF and MB-MDR strategies.

In the case of 2-way interactions, the MB-MDR strategy revealed the rs2619679/rs2928140 (^-4719^A/T / ^2972^C/G; RA/RG) *Rad51* SNP interaction as the one with the strongest association with BrC (Table 9A). The ^-4719^AA/^2972^CG and ^-4719^AT/^2972^CC two-locus genotypes were identified as the “high-risk” genotypes with both of them being more frequent among BrC cases compared to controls (p = 0.071 and p = 0.015, respectively). Analyzing this non-linear interaction effect on BrC as a whole, carriers of these “high-risk” two-locus genotypes were found to be significantly more frequent among cases (cases vs. controls: 10.9% vs. 1.2%), thus indicating an increased risk of BrC among these subjects (OR = 11.3; 95% CI: 2.5–49.5; p<0.005). Moreover, this interaction effect on BrC risk remained statistically significant following the correction for multiple testing using the 10,000 random permutations test (p = 0.0001). Interestingly, the pRF strategy confirmed the results obtained by MB-MDR also identifying the rs2619679/rs2928140 (^-4719^A/T / ^2972^C/G; RA/RG) *Rad51* SNP interaction as being in the strongest association with the BrC/control status (Table 9A). In this strategy, omitting such SNP interaction in RF-based classification model increased the BrC/control classification differential error (ΔE) by 1.81%.

**Table 9:** Epistatic SNP interactions with the strongest association with BrC.: Results of MB-MDR and pRF analysis.

**A**	**MB-MDR**							
	**(RA)** rs2619679	**(RG)** rs2928140	BrC	Controls			Risk group	p
	AA	CG	9 (0.070)	1 (0.006)			High	0.0706
	AT	CC	5 (0.039)	1 (0.006)			High	0.0153
	RA*RG		Cases	Controls	Wald_H_	OR_H_	p_W_	p_10000_
	High		14 (0.109)	2 (0.012)				
	Low/Neutr.		114 (0.891)	180 (0.988)	9.0074	11.3 [2.5–49.5]	0.0027	0.0001
	**pRF**							
			${\bar{E}}_{2}$	${\bar{E}}_{1}$	*ΔE*			
	RA*RG		21,51%	19,70%	1,81%			
**B**	**MB-MDR**							
	**(RA)** rs2619679	**(RB)** rs5030789	**(RG)** rs2928140	Cases	Controls		Risk group	p
	AA	AA	CG	6 (0.047)	1 (0.006)		High	0.0865
	AT	GA	CC	5 (0.039)	1 (0.006)		High	0.0127
	RA*RB*RG		Cases	Controls	Wald_H_	OR_H_	p_W_	p_10000_
	High		11 (0.086)	2 (0.011)				
	Low/Neutr.		117 (0.914)	179 (0.989)	9.0305	8.4 [1.8–38.6]	0.0027	<< 0.0001
	**pRF**							
			${\bar{E}}_{2}$	${\bar{E}}_{1}$	*ΔE*			
	RA*RB*RG		25,24%	21,68%	3,57%			

Results of analysis of associations between **(A)** 2-way or **(B)** 3-way epistatic interactions and BrC are summarized. Absolute (relative) counts of BrC and control subjects are provided for each individual and multi-locus genotype. MB-MDR: p indicates the level of significance for association between individual genotypes, while BrC, Wald_H_, OR_H_, p_W_ and p_10000_ stand for the value of Wald statistic, resulting odds ratio, raw and corrected level of significance for the 2- or 3-way multi-locus genotypes with the strongest association with BrC, respectively. Individuals with given multi-locus genotype of interest were compared against the rest of the individuals, which are considered as reference group. High—high risk of BrC; Low—low risk of BrC; Neutr.–non-informative individuals. pRF: ${\bar{E}}_{2}$ and ${\bar{E}}_{1}$ denote the classification errors for testing dataset with and without the interaction among analyzed predictors, while *ΔE* denotes the differential error for the analyzed interaction.

In the case of 3-way interactions, the MB-MDR strategy revealed the rs2619679/rs5030789/rs2928140 (^-4719^A/T / ^-4601^A/G / ^2972^C/G; RA/RB/RG) *Rad51* SNP triplet as the one with the strongest association with BrC (Table 9B). The ^-4719^AA/^-4601^AA/^2972^CG and ^-4719^AT/^-4601^GA/^2972^CC three-locus genotypes were found to be more frequent among BrC cases compared to controls (p = 0.087 and p = 0.013, respectively) and thus identified as the “high-risk” combination of genotypes. Carriers of these “high-risk” triplets were found to be significantly more frequent among BrC cases (cases vs. controls 8.6% vs. 1.1%) suggesting an increased risk of BrC among these subjects (OR = 8.4; 95% CI: 1.8–38.6; p<0.005) which remained significant following the correction for multiple hypotheses testing (p<<0.0001). The significant association between the rs2619679/rs5030789/rs2928140 (^-4719^A/T / ^-4601^A/G / ^2972^C/G; RA/RB/RG) *Rad51* SNP interaction and the BrC/control status was confirmed also in pRF strategy, according to which involvement of this interaction effect in RF-based classification models improves their differential error (ΔE) by 3.57% (Table 9B). Detailed distribution of subjects with respect to genotypes carried at SNP loci involved in analyzed 2-way or 3-way multi-locus genotypes is presented in S5 Table.

## Discussion

In the hereby presented study we examined the role of genetic variability of two proteins belonging to the HR DSB DNA repair pathway—Rad51 and Xrcc3—as a risk factor for breast cancer in Polish population. In total we investigated 14 common single nucleotide polymorphisms in the genes encoding the above mentioned enzymes, seven SNPs per each protein (rs2619679, rs5030789, rs1801320, rs1801321, rs2619680, rs2619681, and rs2928140 in Rad51; rs1799794, rs45603942, rs861530, rs3212057, rs1799796, rs861539, and rs28903081 in Xrcc3).

In the case of Rad51, no associations with BrC were found for rs2619679, rs1801321, rs2619680, rs2619681, and rs2928140 under any of genetic models assumed in this study. Contrary to this, our study provides some very interesting outcomes concerning the rare **rs5030789** (^-4601^A/G; RB) ^-4601^AA genotype which can be associated with reduced BrC risk under recessive genetic model (OR = 0.5; p<0.05). Outcomes of the analysis under additive genetic model and direct analysis of allele frequencies seem to provide further support in favor of this conclusion suggesting a 30% BrC risk reduction being associated with the variant ^-4601^A allele, yet just beyond the edge of statistical significance (p = 0.06 and p = 0.07, respectively). In our previous study [46] we have found the rare rs5030789 ^-4601^AA genotype to confer some protective effect against head and neck cancer (HNC) among men, thus the present study is yet another report suggesting protective effect of this SNP against cancer. It has to be, however, stated that rs5030789 has not yet been studied in relation to cancer risk by any other group and thus no other reports on its involvement in cancer risk modulation are available. The protective anti-cancer effect of this SNP needs to be thus treated with caution and verified in a larger case-control study. Either way, the rs5030789 in Rad51 seems to be a plausible cancer risk-reducing SNP.

Recent huge meta-analyses involving several tens of thousands of subjects provided solid evidence that the variant **rs1801320** (^135^G/C; RC) ^135^C Rad51 allele localized in the 5’UTR the gene increases the overall risk of cancer [10,24]. The same effect was suggested also in the case of BrC, with the odds ratio of BrC under the recessive genetic model being estimated at 1.7 [24] and 3.3 [10], respectively. The hereby presented outcomes of our study are in line with those cited above, as we have also found the ^135^CC genotype to be more frequent among BrC cases as compared to healthy controls (7.1% vs. 0.8%, respectively), resulting in around 10-times higher odds of BrC among rare homozygotes. Of note might be the fact, that the frequencies and odds reported by our study differ somewhat from the ones reported by earlier study conducted in Polish population, in which this variant genotype was found to be present in as much as almost 70% of BrC cases and 20% of healthy controls [55]. Discrepancies among BrC cases may at least partially be explained by the fact that the study [55] was focused on triple-negative BrC cases only, while we applied no filtering of the BrC cases based on their estrogen, progesterone and HER-2 receptor status. Large difference in frequencies among controls is, however, strange and difficult to be explained. Nevertheless, the study [55] has also provided quite high values of odds ratio for triple-negative BrC being associated with the rare ^135^CC Rad51 genotype (OR = 6.0), analogically to our study.

Of note is the fact that our study seems to provide no evidence in favor of any risk-modifying effect of **rs1801321** (^172^G/T; RD) Rad51 SNP, yet another SNP in Rad51 frequently investigated in relation to cancer risk. This SNP was shown to be located in the P300/CBP transcription factor binding site leading to increased activity of *Rad51* promoter and increased capacity of DSB DNA repair pathway [22,56–58]. Even though the ^172^T allele-containing genotypes were found to be associated with reduced risk of cancer in general [24], their effect on BrC risk could not be unambiguously specified so far. In Polish population, there are only two relatively small studies available so far, delivering results not allowing to draw any conclusion, as well [25,27]. Our study does not provide any evidence useful in clarifying this conundrum, thus the role of ^172^T Rad51 SNP in BrC development remains unresolved.

Concerning the SNPs in Xrcc3 investigated in this study, only **rs3212057** (^10343^G/A, X4) localized in exon 6 of the gene resulting in ^94^Arg/His mismatch mutation can plausibly be associated with BrC. Variant ^10343^A allele was found among the control subjects only, which suggests its protective effect against BrC. Unfortunately, observed level of significance of such effect was close to marginal 0.05 (p = 0.0453) and we were unable to find any supportive outcome in terms of distribution of genotypic frequencies compared under any of the genetic models assumed. Therefore, this outcome needs to be interpreted cautiously mainly due to the fact that this SNP turned out to be quite rare and our study surely lacked the statistical power needed to provide additional reliable outcomes.

Concerning the SNPs in Xrcc3 investigated in this study, only **rs3212057** (^10343^G/A, ^94^Arg/His, X4) in exon 6 of the gene can plausibly be associated with BrC. Variant ^10343^A allele was found among the control subjects only, which suggests its protective effect against BrC. Unfortunately, observed level of significance of such effect was close to marginal 0.05 (p = 0.0453) and we were unable to find any supportive outcome in terms of distribution of genotypic frequencies compared under any of the genetic models assumed. Therefore, this outcome needs to be interpreted cautiously mainly due to the fact that this SNP turned out to be quite rare and our study surely lacked the statistical power needed to provide additional reliable outcomes. Nevertheless, it should be emphasized that the ^94^Arg/His SNP finds itself in the *Xrcc3* gene segment encoding amino acids 63 to 346 of the protein, a region directly involved in Xrcc3/Rad51C heterodimer formation [59]. Owing to crucial role played by this heterodimer in several steps of HR (binding of DNA, resolution of Holiday junctions) [12], a polymorphism affecting its formation may thus influence the capacity of the whole HR DNA repair machinery and play its role in cancer development. Evidence in favor of this hypothesis is, still, rather scarce, as in addition to our previous study in which the Xrcc3 ^94^His allele was found to be associated with increased risk of HNC [46], only four other studies investigating the association between ^94^Arg/His SNP and risk of various cancers exist [60–63]. These studies were, however, conducted in Taiwanese population completely lacking any variability at this locus, so they failed to provide any valuable information on plausible role of rs3212057 in cancer risk modulation. Taken all together, the hereby suggested cancer type- and population-specific association between ^94^Arg/His SNP in Xrcc3 and cancer risk needs to be further verified.

We failed to find any statistically significant association between **rs861539** (^241^Thr/Met) in exon 6 of *Xrcc3* (the most frequently investigated *Xrcc3* SNP) and BrC risk. A huge meta-analysis has shown that this SNP provides slight but statistically significant BrC risk increase [64], mainly due to altered protein function, increased genetic instability and DNA DSB accumulation [23]. Recently performed studies on Polish population, however, failed to confirm such effect on either unselected or triple-negative BrC [32,33]. It has to be, however stated, that these two Polish studies, alike the hereby presented one, were relatively small in their sizes (up to 200 BrC subjects only), thus the effect revealed by a meta-analysis involving almost 10,000 BrC cases may have not simply been provable.

For those SNPs identified in single-site analyses as significantly associated with BrC risk, we further analyzed the effect of all possible combinations of respective high-risk genotypes on BrC. Based on obtained outcomes, it is obvious that the relationship between BrC risk and the number of possessed high-risk genotypes is not linear nor additive. One may, however notice that the combinations containing Rad51 ^-4601^GG or ^-4601^GA (RB) seem to confer a higher BrC risk compared to the risk associated with the RB SNP itself, while, on the other hand, the combinations containing Rad51 ^135^CC (RC) SNP tend not to present such distinct one-directional change of BrC risk when compared to RC-only-associated BrC risk. Interpretation of how the resultant disease risk changes due to such combinations is even more difficult due to our inability to exactly count the BrC risk associated with the Xrcc3 ^10343^G/A (X4) SNP (due to zero BrC cases carrying the low-risk genotype). Even though it seems unable to draw any further conclusions on the influence of combinations of Rad51 and Xrcc3 high-risk genotypes on BrC risk, it is worth mentioning that in spite of the hereby presented outcomes being obtained based on relatively small number of subjects (see Table 7) in an analysis adjusted to confounders, the outcomes related to genotype combinations are characterized with considerably higher levels of significance compared to *p*-levels obtained in respective single-site analyses. It thus clearly shows that analyzing SNP combinations instead of individual SNPs may lead to increased statistical power and that it may indeed be the way of how to move forward in search for links between genetic variability and complex diseases, especially in the case of SNPs within closely related proteins from a given pathway.

In haplotype analysis, none of the 6 haplotypes reconstructed within the rs1801321-rs2619680- rs2619681 (i.e. RD-RE-RF) LD block in *Rad51* were found to be associated with the BrC risk, which seems to be in line with the results of above discussed single-site analyses of these three SNPs. Contrary to this, outcomes of haplotype analysis for rs5030789 (RB) and rs1801320 (RC), which were found to be associated with BrC risk in single-site and SNP combinations analyses, however suggest that these SNPs are not part of any LD block and most probably recombine during meiotic chromatid segregation and modulate the BrC risk independently of each other. In the case of Xrcc3, rs3212057 (X4), significantly associated with BrC risk in single-site analysis, was not the part of any of two LD blocks identified within the *Xrcc3* gene. Instead, the ^4541^A/^9685^A haplotype within the LD block spanning from rs1799794 (X1) to rs861530 (X3) was found to confer a significantly lower BrC risk. Such an interesting observation of two SNPs not associated with BrC risk in single-site analyses revealing significant risk modulating effect in haplotype analysis may simply indicate that the possession of certain variant in one SNP locus might be not enough to impose the risk-modulating effect, and that certain “configuration” of two or several other SNPs is required for the effect to take place. Nevertheless, the haplotype analyses in relation to cancer risk are still very scarce, rendering thus discussion of such outcomes quite challenging. The very few studies analyzing possible cancer risk-modifying effects of *Rad51* and/or *Xrcc3* haplotypes are, unfortunately, related to different type of cancers [46,65].

In addition to above described analyses, we investigated the effect of nonlinear SNP-SNP (i.e. epistatic) interactions between selected SNPs on the risk of BrC as well. Pure nonlinear epistatic interactions are believed to affect functionality of ternary and quaternary structures involved in specific biological processes without any directly observable main effects of interacting SNPs [53]. Since Xrcc3 and Rad51C were shown to form a heterodimer which directly interacts with Rad51 facilitating the functionality of HR DNA DSB repair machinery, we hypothesized that epistatic interactions between these three proteins might at least partially influence the system’s DNA repair capacity and affect the resultant BrC risk. However, due to the fact that conventional statistical methods (linear regression, logistic regressions, chi-square tests, etc.) turned out to be ineffective and not able to deal with some very specific challenges faced in such kind of analyses (such as the so-called curse of dimensionality, large computational burden, etc.), machine learning techniques have recently became increasingly implemented in order to uncover associations between complex diseases (including cancer) and such otherwise “hidden” interactions. These techniques are becoming a hallmark of the so-called post-GWAS era, in which it is known that risk profiles generated by common low and/or moderate susceptibility loci put together in a simple additive model provide only limited usefulness with respect to complex diseases risk prediction [66,67].

To elucidate plausible effects of nonlinear epistatic interactions between Xrcc3, Rad51 and Rad51C SNPs on BrC risk, two machine learning techniques, presenting two different approaches were used: MB-MDR which is based on a simple model (logistic regression), and pRF built upon a model-free CART analysis. Quite surprisingly, despite different approaches, both these methods pointed out the same interaction as the one with the strongest effect on BrC risk, irrespectively of whether 2-way or 3-way interactions were considered. In the case of 2-way interactions, a simple yet interesting model was proposed by MB-MDR, according to which the highest BrC risk is dependent on inheriting just one heterozygous genotype from two different loci—either the rs2619679 (RA) ^-4719^AT or the rs2928140 (RG) ^2972^CG, but not both. We cannot currently provide any biologically plausible interpretation of this outcome, as details of molecular interaction among Rad51-family proteins and/or DNA are still unknown. We can only speculate that these SNPs may be localized in gene regions crucial with respect to biological/functional properties of Rad51. Of note is, however, the fact that this interactional effect on BrC risk has been identified as the pure epistatic effect, with no main effects observable for neither rs2619679 (RA) or rs2928140 (RG) in single-site analyses, nor they have been suggested as being involved in haplotypes affecting the BrC risk. According to pRF outcomes, involving this interaction in a classification model results in increased classification “correctness” by some 2% (Table 9), which doesn’t seem to be a considerably high value, but it is quite astonishing when we realize that it is a result of allowing the model for a combination of just 2 out of some 10 million SNPs found in human genome. It is just as striking that subjects belonging to high-risk group identified within this model are conferred with quite considerable BrC risk increase (OR = 11.3), which furthermore turned out to be highly statistically significant (p_10000_ = 0.0001, corrected for multiple hypotheses testing). Of course, this model cannot be yet considered as clinically useful, as more studies aimed to verify and validate its clinical relevancy in larger experimental setup involving also higher-order interaction models are needed. The importance of the order of such interaction is argued for by the fact that involving 3-way epistatic interactions among SNPs in a classification model almost doubled the gain in classification correctness in this study. Here, both methods indicated the rs2619679/rs5030789/rs2928140 (i.e. RA/RB/RG) interaction as the one with the strongest association to BrC. Adding the rs5030789 (RB) ^-4601^G/A SNP into previously identified RA/RG best 2-way interaction provides basically more detailed specification of the high-risk group, showing that high risk of BrC in subjects carrying either rs2619679 (RA) ^-4719^AT or the rs2928140 (RG) ^2972^CG genotypes is dependent on co-occurrence of either rs5030789 (RB) ^-4601^GA or the rs5030789 (RB) ^-4601^AA genotypes, respectively (Table 9). It all somehow suggests that it takes two more rare RB alleles (i.e. ^-4601^AA) for the ^-4719^AA/^2972^CG carriers to exert the BrC risk-increasing effect, while only one such RB allele (i.e. ^-4601^GA) is sufficient for BrC risk increase among ^-4719^AT/^2972^CC carriers. Even though the high-risk group was narrowed based on additional third locus, it still retained its relatively high increase of BrC risk (OR = 8.5) as well as its high level of statistical significance (p_10000_ = 0.0001 corrected for multiple hypotheses testing). The correctness of classification model was also improved by almost 3.6%. Nevertheless, the biological/functional background of such effects are unknown.

To sum all outcomes up, Table 10 briefly highlights all outcomes obtained in the study. Of note here is especially the rs5030789 (RB) SNP, for which evidence suggesting its involvement in BrC risk modulation was provided by four out of five different analytical methods (single-site analysis, VIMP-based ranking, MB-MDR and pRF). Moreover, it was the only SNP found to be associated with BrC in both single-site and epistatic interaction analysis, although the predictions of this SNP’s effect on BrC risk seems to be contradictory. It might be a bit puzzling to understand how both protective and detrimental effects on disease risk may be exerted by a single SNP, nevertheless, it has to be kept in mind, that, by definition, epistatic effects based on interaction with other SNPs may lead to completely different effects compared to those observed for individual interacting SNPs. Considering the fact that this SNP is placed within the crucial player of the whole HR DSB DNA repair machinery, it seems as a strong rationale for rs5030789 (RB) being indeed of importance with respect to BrC development, although complete understanding of its role requires further studies.

**Table 10:** Summary of inferred effects of individual SNPs on BrC risk.

#rs	code	gene	genotype/allele	effect on BrC risk
*Main effect*				
rs5030789	RB	*Rad51*	^-4601^AA	protective
rs1801321	RC	*Rad51*	^135^CC	detrimental
rs1799794	X1	*Xrcc3*	^4541^A^a^	protective
rs861530	X3	*Xrcc3*	^9685^A^a^	protective
rs3212057	X4	*Xrcc3*	^10343^GA	protective
*Epistatic effect*				
rs2619679	RA	*Rad51*	^-4719^AA^b^^,^^d^	detrimental
			^-4719^AT^c^^,^^d^	detrimental
rs5030789	RB	*Rad51*	^-4601^AA^b^	detrimental
			^-4601^GA^c^	detrimental
rs2928140	RG	*Rad51*	^2972^CG^b^^,^^d^	detrimental
			^2972^CC^c^^,^^d^	detrimental
*Unclear effect*				
rs1801321	RD	*Rad51*	-	unclear ^e^
*No effect*				
rs2619680	RE	*Rad51*	-	-
rs2619681	RF	*Rad51*	-	-
rs45603942	X2	*Xrcc3*	-	-
rs1799796	X5	*Xrcc3*	-	-
rs861539	X6	*Xrcc3*	-	-
rs28903081	X7	*Xrcc3*	-	-

Summary of effects of individual SNPs on BrC risk inferred based on statistically significant outcomes of analyses conducted in the study. *Main* or *epistatic* effects were inferred if statistically significant outcomes were revealed by conventional (single-site, SNP combinations, haplotype) or ML-based analyses, respectively. Of note is the rs5030789 (RB) SNP in *Rad51*, for which protective main effect as well as detrimental epistatic effect was predicted.

^a^ as part of the risk-reducing X1/X3 haplotype;

^b^ as part of the risk-increasing ^-4719^AA/^-4601^AA/^2927^CG three-locus genotype;

^c^ as part of the risk-increasing ^-4719^AT/^-4601^GA/^2927^CC three-locus genotype;

^d^ pure epistatic effect with no observable main effects;

^e^ statistically significant 2^nd^ place in the VIMP-based ranking with no significant outcomes in any of conventional or ML-based methods.

The rare rs1801320 (RC) genotype, on the other hand, seems to exert detrimental effect on BrC risk and the conclusion in based on outcomes of single-site analysis and high place in the VIMP-based ranking (3^rd^ or 4^th^ place). Such combination of outcome suggests rather considerable main effect of this genotype on BrC risk, without any additional epistatic effects, as simple VIMP-based rankings cannot distinguish between main and interactional effects [68]. Importance of this SNP with respect to BrC risk seems to be, however, in line with outcomes reported previously by meta-analyses, in which rs1801320 ^135^C allele was found to be associated with significant cancer (including BrC) risk increase [10,24].

Outcomes for rs3212057 (X4) also suggest that heterozygous ^10343^GA genotype in this locus may protect against BrC, as some indications of such effect were found in single-site analysis and further supported by statistically significant yet rather low (11^th^ and 12^th^ place) place in VIMP-based ranking. This conclusion should be, however, interpreted with caution as the amount of currently available evidence on involvement of rs3212057 in carcinogenesis is still limited.

Outcomes for rs1799794 (X1) and rs861530 (X3) (statistically significant protective effect of X1/X3 haplotype), rs1801321 (RD) (2^nd^ place in VIMP-based rankings with statistically significant VIMP values) as well as rs2619679 (RA) and rs2928140 (RG) (both involved in risk-increasing three-locus genotype revealed by both MB-MDR and pRF) these are relatively novel outcomes definitely requiring further verification (Table 10).

It is worth mentioning that the analysis based on machine learning techniques pointed out an interaction between SNPs localized within the only one gene. This conclusion is further supported by the VIMP-based ranking, where *Rad51* SNPs took four out of five best ranks. Although this underlines the importance of *Rad51* SNPs with respect to BrC, we cannot say whether it would be so also in the case of higher–order interactions.

Among minor limitations our study admittedly suffers from, one has to mention the lack of proper case-control matching and limited study size. While the lack of proper matching was partially solved by considering certain subjects’ characteristics as confounders in each analysis enabling to do so, the limited study size was balanced out by employing novel machine learning approach specifically designed to overcome such limitation. Nevertheless, the low study size still prevented us from being able to verify our outcomes in a validation group, an approach often used as a golden standard with the hereby used techniques. It is also possible that some other machine learning techniques could have led to slightly different outcomes, nevertheless, the goal of this study was to investigate the role of genetic variability of Rad51 family members in BrC development and compare outcomes obtained by conventional analytical methods to those obtained by the most popular novel machine learning techniques used in the field, rather than provide comprehensive comparison of all possible approaches. Last but not least, data on some of well-known BrC risk factors interacting with HR DSB DNA repair pathway (such as *BRCA1* and *BRCA2* mutations, alcohol consumption) would possibly increase the significance of outcomes and help better understand the above discussed relationships, but such data were unavailable. Either way, we still argue that our study provides some valuable novel outcomes which may successfully provide clues for future fruitful research.

To sum all up, our study provides evidence that the genetic variability of Xrcc3 and Rad51 may be of relevance with respect to BrC risk modulation. Especially the rs5030789 ^-4601^G/A Rad51 SNP seems to be of importance in this regard, as it was found to independently predict the disease risk as well as to co-participate (together with specific rs2619679 ^-4719^A/T and rs2928140 ^2972^C/G genotypes) in a BrC risk-modulating epistatic interactions, suggesting its possible complex role in BrC development. Important roles in BrC risk modulation were suggested also for rs1801320 ^135^CC Rad51 genotype and rs3212057 ^10343^A (^94^His) Xrcc3 allele.

## Supporting information

S1 Fig Multiple sequence alignment of Rad51, Xrcc3 and Rad51C amino acid sequences across 16 different species. The species involved in sequence alignment include selected representatives of mammals, birds, amphibians, fish, fungi and protozoa. The sequences have been downloaded from UniProt database (The UniProt Consortium. Nucleic Acids Res. 2019;47(D1):D506) and have been aligned using ClustalOmega algorithm (Sievers F, et al. Mol. Syst. Biol. 2011;11(7):539) implemented in *msa* R package (Bodenhofer U, et al. Bioinformatics 2015;31(24):3997) with default parameters. Blue-shaded letters indicate that the amino acid at given position is conserved in the majority (i.e. >50%) of aligned sequences. Red annotated rectangles indicate the position of mismatch SNPs, i.e. rs3212057 (X4), rs861539 (X6) and rs28903081 (X7) in Xrcc3. X4 is localized in a relatively invariant (conserved in >56% of involved species) residue, while X6 and X7 are localized in variable residues. All remaining SNPs were localized in non-coding regions and are thus not shown within aligned amino acid sequences. From the alignment itself it is obvious, that Rad51 is highly conserved among all involved species, while Xrcc3 and Rad51C are relatively highly conserved among chordates and vertebrate species.
(PDF)

S1 File Thorough description of algorithms used in analyses based on machine learning techniques. This supporting information file provides the full and detailed description of the RF-based strategy used to analyze simple associations between SNP predictors and BrC (Part A), as well as algorithms used in analysis of epistatic interactions between SNPs and BrC (Part B: pRF; Part C: MB-MDR).
(DOCX)

S1 Table Dataset. The table provides raw dataset including data for analyzed SNPs as well as for each individual’s age and smoking status (binary-coded) used in analyses as covariates.
(XLS)

S2 Table Results of the systematic examination of the impact of crucial parameters affecting the resultant RF ability to accurately predict the BrC/control status. The table shows the results of the first step of the strategy (searching for the best RF models) employed to rank all analyzed SNPs with respect to their ability to accurately predict the BrC/control status.
(XLS)

S3 Table Basic characteristics of RFs used in analyses aimed to obtain the VIMP-based ranking of predictors and in RF-based analysis of epistatic interactions. The tables present the classification errors obtained by classifying all the enrolled subject using the RF-based models selected as the best ones for subsequent RF-based analyses.
(XLS)

S4 Table Bootstrap estimates of distribution of the VIMP-based ranks of analyzed predictors. The table presents the results of the validation of obtained VIMP-based ranking of SNPs, which was performed by bootstrapping the whole ranking procedure 10,000 times using the same RF models.
(XLS)

S5 Table Distribution of BrC and control subjects with respect to genotypes carried at SNP loci involved in analyzed 2-way or 3-way multi-locus genotype. (XLS)
