# Supplementary material for: Rad51 paralogs and the risk of unselected breast cancer: A case-control study
Source: PLoS One. 2020 Jan 6;15(1):e0226976. doi: 10.1371/journal.pone.0226976 (PMC6944361; doi:10.1371/journal.pone.0226976)
Supplement: S1 Fig — The species involved in sequence alignment include selected representatives of mammals, birds, amphibians, fish, fungi and protozoa. The sequences have been downloaded from UniProt database (The UniProt Consortium. Nucleic Acids Res. 2019;47(D1):D506) and have been aligned using ClustalOmega algorithm (Sievers F, et al. Mol. Syst. Biol. 2011;11(7):539) implemented in msa R package (Bodenhofer U, et al. Bioinformatics 2015;31(24):3997) with default parameters. Blue-shaded letters indicate that the amino acid at given position is conserved in the majority (i.e. >50%) of aligned sequences. Red annotated rectangles indicate the position of mismatch SNPs, i.e. rs3212057 (X4), rs861539 (X6) and rs28903081 (X7) in Xrcc3. X4 is localized in a relatively invariant (conserved in >56% of involved species) residue, while X6 and X7 are localized in variable residues. All remaining SNPs were localized in non-coding regions and are thus not shown within aligned amino acid sequences. From the alignment itself it is obvious, that Rad51 is highly conserved among all involved species, while Xrcc3 and Rad51C are relatively highly conserved among chordates and vertebrate species. (PDF) [file pone.0226976.s001.pdf]

Rad51

```
logo
Homo sapiens              ........................................................MAMQMQLEAN.ADTSVEEESFG.PQPISRLEQCGINANDVKKLE   42
Pan troglodytes           ........................................................MAMQMQLEAN.ADTSVEEESFG.PQPISRLEQCGINANDVKKLE   42
Macaca mulatta            ........................................................MAMQMQLEAN.ADTSVEEESFG.PQPISRLEQCGINANDVKKLE   42
Tursiops truncatus        ........................................................MAMQMQLEAN.ADTSVEEESFG.PQPISRLEQCGIHANDVKKLE   42
Bos taurus                ........................................................MAMQMQLETN.ADTLWKKKGFG.PQPISRLEQCGINANDVKKLE   42
Canis lupus f.            ........................................................MAMQMQLEAN.ADTSVEEESFG.PQPISRLEQCGINANDVKKLE   42
Felis catus               ........................................................MAMQMQLEAN.ADTSVEEESFG.PQPISRLEQCGINASDVKKLE   42
Rattus norvegicus         ........................................................MAMQMQLEAN.ADTSVEEESFG.PQPISRLEQCGINANDVKKLE   42
Mus musculus              ........................................................MAMQMQLEAS.ADTSVEEESFG.PQPISRLEQCGINANDVKKLE   42
Cricetulus griseus        ........................................................MAMQMQLEAN.ADTSVEEESFG.PQPISRLEQCGISANDVKKLE   42
Gallus gallus             ........................................................MAMQVQFEAS.TDTSAEEESFG.PEPISRLEQCGINANDVKKLE   42
Xenopus laevis            ........................................................MAMQAHYEAE....ATEEEHFG.PQAISRLEQCGINANDVKKLE   39
Danio rerio               .........................................................MAMRNASRVEVEAEVEEENFG.PQPVSRLEQSGISSSDIKKLE   43
Drosophila melanogaster   ............................................................MEKLTNVQAQQEEEEEE.GPLSVTKLIGGSITAKDIKLLQ   39
Saccharomyces cerevisiae  MSQVQEQHISESQLQYGNGSLMSTVPADLSQSVVDGNGNGSSEDIEATNGSGDGGGLQEQAEAQGEMEDEAYDEAALGSFVPIEKLQVNGITMADVKKLR  100
Trichomonas vaginalis     ..................................................................MDTEVAQEGAAG.PLLIQTLEKQGIAAGDIKKLQ   33

logo
Homo sapiens              EAGFHTVEAVAYAPKKELINIKGISEAKADKILAEAAKLVPMGFTTATEFHQRRSEIIQITTGSKELDKLLQGGIETGSITEMFGEFRTGKTQICHTLAV  142
Pan troglodytes           EAGFHTVEAVAYAPKKELINIKGISEAKADKILAEAAKLVPMGFTTATEFHQRRSEIIQITTGSKELDKLLQGGIETGSITEMFGEFRTGKTQICHTLAV  142
Macaca mulatta            EAGFHTVEAVAYAPKKELINIKGISEAKADKILAEAAKLVPMGFTTATEFHQRRSEIIQITTGSKELDKLLQGGIETGSITEMFGEFRTGKTQICHTLAV  142
Tursiops truncatus        EAGFHTVEAVAYAPKKELINIKGISEAKADKILTEAAKLVPMGFTTATEFHQRRSEIIQITTGSKELDKLLQGGIETGSITEMFGEFRTGKTQICHTLAV  142
Bos taurus                EAGFHTVEAVAYAPKKELINIKGISEAKADKILTEAAKLVPMGFTTATEFHQRRSEIIQITTGSKELDKLLQGGIETGSITEMFGEFRTGKTQICHTLAV  142
Canis lupus f.            EAGFHTVEAVAYAPKKELISIKGISEAKADKILTEAAKLVPMGFTTATEFHQRRSEIIQITTGSKELDKLLQGGIETGSITEMFGEFRTGKTQICHTLAV  142
Felis catus               EAGFHTVEAVAYAPKKELINIKGISEAKADKILTEAAKLVPMGFTTATEFHQRRSEIIQITTGSKELDKLLQGGIETGSITEMFGEFRTGKTQICHTLAV  142
Rattus norvegicus         EAGYHTVEAVAYAPKKELINIKGISEAKADKILAEAAKLVPMGFTTATEFHQRRSEIIQITTGSKELDKLLQGGIETGSITEMFGEFRTGKTQICHTLAV  142
Mus musculus              EAGYHTVEAVAYAPKKELINIKGISEAKADKILTEAAKLVPMGFTTATEFHQRRSEIIQITTGSKELDKLLQGGIETGSITEMFGEFRTGKTQICHTLAV  142
Cricetulus griseus        EAGFHTVEAVAYAPKKELINIKGISEAKADKILAEAAKLVPMGFTTATEFHQRRSEIIQITTGSKELDKLLQGGIETGSITEMFGEFRTGKTQICHTLAV  142
Gallus gallus             EAGYHTVESVAHAPKKELLNIKGISEAKADKILAEAAKLVPMGFTTATEFHQRRSEIIQITTGSKELDKLLQGGIETGSITELFGEFRTGKTQLCHTLAV  142
Xenopus laevis            EAGFHTVEAVAYAPKKELLNIKGISEAKAEKILAEAAKLVPMGFTTATEFHQRRSEIIQISTGSKELDKLLQGGVETGSITEMFGEFRTGKTQLCHTLAV  139
Danio rerio               DGGFHTVEAVAYAPKKELLNIKGISEAKADKILTEAAKMVPMGFTTATEFHQRRAEIIQISTGSKELDKLLQGGIETGSITEMFGEFRTGKTQLCHTLAV  143
Drosophila melanogaster   QASLHTVESVANATKKQLMAIPGLGGGKVEQIITEANKLVPLGFLSARTFYQMRADVVQLSTGSKELDKLLGGGIETGSITEIFGEFRCGKTQLCHTLAV  139
Saccharomyces cerevisiae  ESGLHTAEAVAYAPRKDLLEIKGISEAKADKLLNEAARLVPMGFVTAADFHMRRSELICLTTGSKNLDTLLGGGVETGSITELFGEFRTGKSQLCHTLAV  200
Trichomonas vaginalis     EAGYNTVESVAFTTKKQLITVKGISEAKADKIMAAAAQLVPMGFASASDYNQIRKEMVYLTTGSTELDKLLGGGIETGSVTEIFGEFRTGKTQLCHTLAV  133

logo
Homo sapiens              TCQLPIDRGGGEGKAMYIDTEGTFRPERLLAVAERYGLSGSDVLDNVAYARAFNTDHQTQLLYQASAMMVESRYALLIVDSATALYRTDYSGRGELSARQ  242
Pan troglodytes           TCQLPIDRGGGEGKAMYIDTEGTFRPERLLAVAERYGLSGSDVLDNVAYARAFNTDHQTQLLYQASAMMVESRYALLIVDSATALYRTDYSGRGELSARQ  242
Macaca mulatta            TCQLPIDRGGGEGKAMYIDTEGTFRPERLLAVAERYGLSGSDVLDNVAYARAFNTDHQTQLLYQASAMMVESRYALLIVDSATALYRTDYSGRGELSARQ  242
Tursiops truncatus        TCQLPIDRGGGEGKAMYIDTEGTFRPERLLAVAERYGLSGSDVLDNVAYARGFNTDHQTQLLYQASAMMVESRYALLIVDSATALYRTDYSGRGELSARQ  242
Bos taurus                TCQLPIDRGGGEGKAMYIDTEGTFRPERLLAVAERYGLSGSDVLDNVAYARGFNTDHQTQLLYQASAMMVESRYALLIVDSATALYRTDYSGRGELSARQ  242
Canis lupus f.            TCQLPIDRGGGEGKAMYIDTEGTFRPERLLAVAERYGLSGSDVLDNVAYARGFNTDHQTQLLYQASAMMVESRYALLIVDSATALYRTDYSGRGELSARQ  242
Felis catus               TCQLPIDRGGGEGKAMYIDTEGTFRPERLLAVAERYGLSGSDVLDNVAYARGFNTDHQTQLLYQASAMMVESRYALLIVDSATALYRTDYSGRGELSARQ  242
Rattus norvegicus         TCQLPIDRGGGEGKAMYIDTEGTFRPERLLAVAERYGLSGSDVLDNVAYARGFNTDHQTQLLYQASAMMVESRYALLIVDSATALYRTDYSGRGELSARQ  242
Mus musculus              TCQLPIDRGGGEGKAMYIDTEGTFRPERLLAVAERYGLSGSDVLDNVAYARGFNTDHQTQLLYQASAMMVESRYALLIVDSATALYRTDYSGRGELSARQ  242
Cricetulus griseus        TCQLPIDRGGGEGKAMYIDTEGTFRPERLLAVAERYGLSGSDVLDNVAYARGFNTDHQTQLLYQASAMMVESRYALLIVDSATALYRTDYSGRGELSARQ  242
Gallus gallus             TCQLPIDRGGGEGKAMYIDTEGTFRPERLLAVAERYGLSGSDVLDNVAYARGFNTDHQTQLLYQASAMMAESRYALLIVDSATALYRTDYSGRGELSARQ  242
Xenopus laevis            TCQLPIDRGGGEGKAMYIDTEGTFRPERLLAVAERYGLSGSDVLDNVAYARAFNTDHQTQLLYQASAMMAESRYALLIVDSATALYRTDYSGRGELSARQ  239
Danio rerio               TCQLPIDQGGGEGKAMYIDTEGTFRPERLLAVAERYGLVGSDVLDNVAYARAFNTDHQTQLLYQASAMMTESRYALLIVDSATALYRTDYSGRGELSARQ  243
Drosophila melanogaster   TCQLPISQKGGEGKCMYIDTENTFRPERLAAIAQRYKLNESEVLDNVAFTRAHNSDQQTKLIQMAAGMLFESRYALLIVDSAMALYRSDYIGRGELAARQ  239
Saccharomyces cerevisiae  TCQIPLDIGGGEGKCLYIDTEGTFRPVRLVSIAQRFGLDPDDALNNVAYARAYNADHQLRLLDAAAQMMSESRFSLIVVDSVMALYRTDFSGRGELSARQ  300
Trichomonas vaginalis     TCQLPIENGGGQGKCLWIDTEGTFRPERIIPIAQRFGLDPDDVMDNIAYARAYNSDHQSQLLVTACAMMAEAHYALLIVDSSTNLYRTDYSGRGELSARQ  233

logo
Homo sapiens              MHLARFLRMLLRLADEFGVAVVITNQVVAQVDGAAMFAADPKKPIGGNIIAHASTTRLYLRKGRGETRICKIYDSPCLPEAEAMFAINADGVGDAKD...  339
Pan troglodytes           MHLARFLRMLLRLADEFGVAVVITNQVVAQVDGAAMFAADPKKPIGGNIIAHASTTRLYLRKGRGETRICKIYDSPCLPEAEAMFAINADGVGDAKD...  339
Macaca mulatta            MHLARFLRMLLRLADEFGVAVVITNQVVAQVDGAAMFAADPKKPIGGNIIAHASTTRLYLRKGRGETRICKIYDSPCLPEAEAMFAINADGVGDAKD...  339
Tursiops truncatus        MHLARFLRMLLRLADEFGVAVVITNQVVAQVDGAAMFAADPKKPIGGNIIAHASTTRLYLRKGRGETRICKIYDSPCLPEAEAMFAINADGVGDAKD...  339
Bos taurus                MHLARFLRMLLRLADEFGVAVVITNQVVAQVDGAAMFAADPKKPIGGNIIAHASTTRLYLRKGRGETRICKIYDSPCLPEAEAMFAINADGVGDAKD...  339
Canis lupus f.            MHLARFLRMLLRLADEFGVAVVITNQVVAQVDGAAMFAADPKKPIGGNIIAHASTTRLYLRKGRGETRICKIYDSPCLPEAEAMFAINADGVGDAKD...  339
Felis catus               MHLARFLRMLLRLADEFGVAVVITNQVVAQVDGAAMFAADPKKPIGGNIIAHASTTRLYLRKGRGETRICKIYDSPCLPEAEAMFAINADGVGDAKD...  339
Rattus norvegicus         MHLARFLRMLLRLADEFGVAVVITNQVVAQVDGAAMFAADPKKPIGGNIIAHASTTRLYLRKGRGETRICKIYDSPCLPEAEAMFAINADGVGDAKD...  339
Mus musculus              MHLARFLRMLLRLADEFGVAVVITNQVVAQVDGAAMFAADPKKPIGGNIIAHASTTRLYLRKGRGETRICKIYDSPCLPEAEAMFAINADGVGDAKD...  339
Cricetulus griseus        MHLARFLRMLLRLADEFGVAVVITNQVVAQVDGAAMFTADPKKPIGGNIIAHASTTRLYLRKGRGETRICKVYDSPCLPEAEAMFAINADGVGDAKD...  339
Gallus gallus             MHLARFLRMLLRLADEFGVAVVITNQVVAQVDGAAMFAADPKKPIGGNIIAHASTTRLYLRKGRGETRICKIYDSPCLPEAEAMFAINADGVGDAKE...  339
Xenopus laevis            MHLARFLRMLLRLADEFGVAVVITNQVVAQVDGAAMFAADPKKPIGGNIIAHASTTRLYLRKGRGETRICKIYDSPCLPEAEAMFAINADGVGDAKD...  336
Danio rerio               GHLGRFLRMLLRLADEFGVAVVITNQVVAQVDGAAMFSADPKKPIGGNILAHASTTRLYLRKGRGETRICKIYDSPCLPEAEAMFAINADGVGDAKD...  340
Drosophila melanogaster   NHLGLFLRMLQRLADEFGVAVVITNQVTASLDGAPGM.FDAKKPIGGHIMAHSSTTRLYLRKGKGETRICKIYDSPCLPESEAMFAILPDGIGDARES..  336
Saccharomyces cerevisiae  MHLAKFMRALQRLADQFGVAVVVTNQVVAQVDGGMAFNPDPKKPIGGNIMAHSSTTRLGFKKGKGCQRLCKVVDSPCLPEAECVFAIYEDGVGDPREEDE  400
Trichomonas vaginalis     MHLGQFLRNIQRLADEYGIAVVITNQVVAQVDGAAMFVSDPKKPIGGNIMAHASQTRLSLRKGRGETRVCKIYDSPSLPENEATFQITNGGITDAE....  329
```

X non-conserved

X ≥50% conserved

Xrcc3

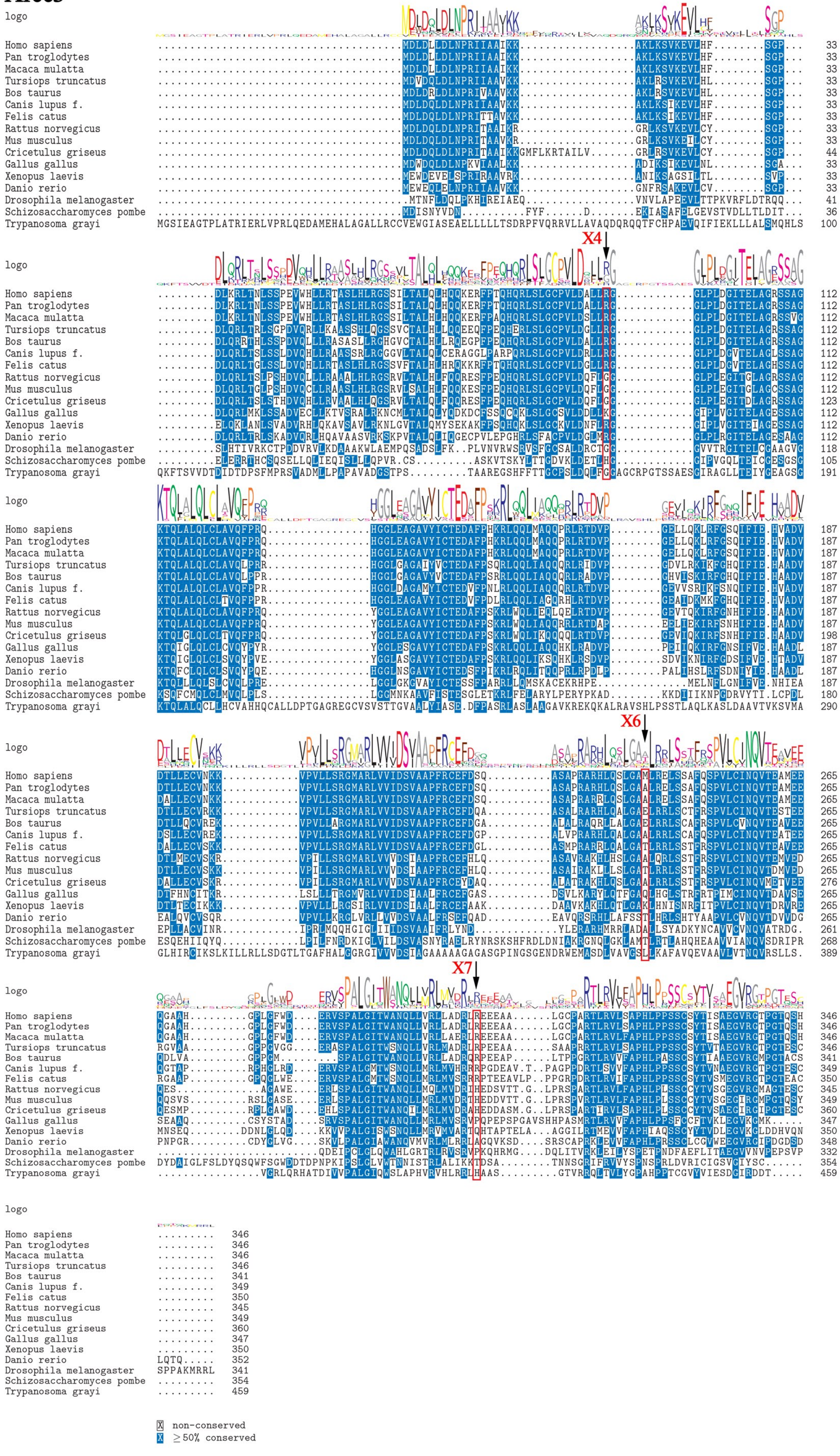
logo
Homo sapiens
Pan troglodytes
Macaca mulatta
Tursiops truncatus
Bos taurus
Canis lupus f.
Felis catus
Rattus norvegicus
Mus musculus
Cricetulus griseus
Gallus gallus
Xenopus laevis
Danio rerio
Drosophila melanogaster
Schizosaccharomyces pombe
Trypanosoma grayi
X4
X6
X7
non-conserved
≥50% conserved

Rad51C

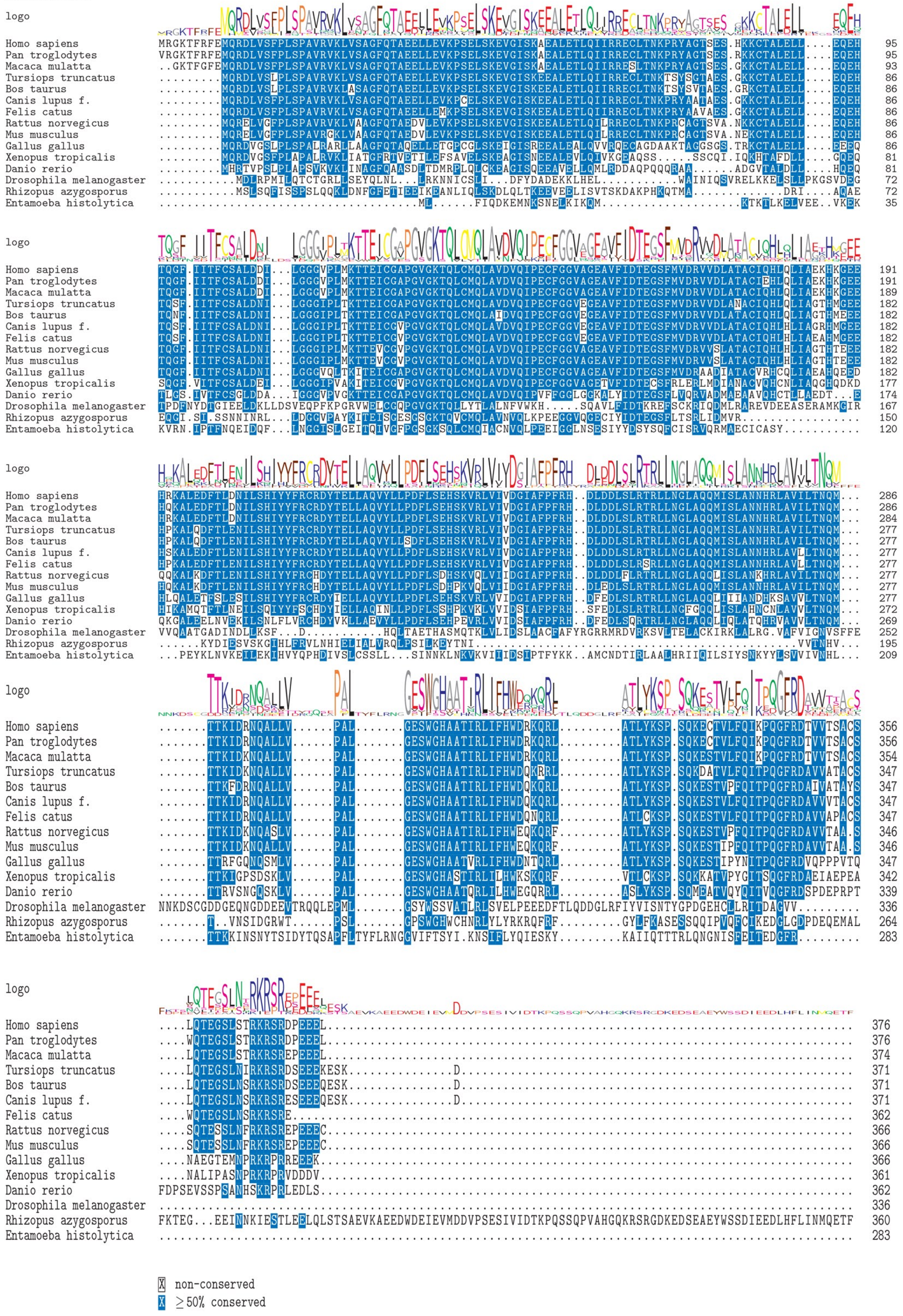
logo
Homo sapiens MRGKTFRFEMQRDLVSFPLSPAVRVKLVSAGFQTAEELLEVKPSELSKEVGISKAEALETLQIIRRECLTNKPRYAGTSES.HKKCTALELL....EQEH 95
Pan troglodytes VRGKTFRFEMQRDLVSFPLSPAVRVKLVSAGFQTAEELLEVKPSELSKEVGISKAEALETLQIIRRECLTNKPRYAGTSES.RKKCTALELL....EQEH 95
Macaca mulatta ..GKTFGFEMQRDLVSFPLSPAVRVKLVSAGFQTAEELLEVKPSELSKEVGISKAEALETLQIIRRECLTNKPRYAGTSES.GKKCTALELL....EQEH 93
Tursiops truncatus .........MQRDLVSLPLSPAVRVKLVSAGFQTAEELLEVKPSELSKEVGISKEEALETLQIIRRESLTNKTSYSGTAES.GKKCTALELL....EQEH 86
Bos taurus .........MQRDLVSLPLSPAVRVKLASAGFQTAEELLEVKPSELSKEVGISKEEALETLQIIRRECLTNKTSYSVTAES.GRKCTALELL....EQEH 86
Canis lupus f. .........MQRDLVSFPLSPAVRVKLVSAGFQTAEELLEVKPCELSKEVGISKEEALETLQIIRRECLTNKPRYAATAES.GKKCTALELL....EQEH 86
Felis catus .........MQRDLVSFPLSPAVRVKLVSAGFQTAEELLEMKPSELSKEVGISKEEALETLQIIRRECLTNKPRYAAVAES.GKKCTALELL....EQEH 86
Rattus norvegicus .........MQRELVGFPLSPAVRVKLVAAGFQTAEDVLEVKPSELSKEVGISKEEALETLQILRRECLTNKPRCAGTSVA.NKKCTALELL....EQEH 86
Mus musculus .........MQRELVGFPLSPAVRGKLVAAGFQTAEDVLEVKPSELSKEVGISKEEALETLQILRRECLTNKPRCAGTSVA.NEKCTALELL....EQEH 86
Gallus gallus .........MQRDVGSLPLSPALRARLLAAGFQTAQELLETGPCGLSKEIGISREEALEALQVVRQECAGDAAKTAGGSGS.TRKCTALELL....EEEQ 86
Xenopus tropicalis .........MQRDVGSFPLAPALRVKLIATGFRTVETILEFSAVELSKEAGISNEEALEVLQIVKGEAQSS.....SSCQI.IQKHTAFDLL....GQEQ 81
Danio rerio .........MHRTVPSLPLAPSVKVKLINAGFQAASDLTDMRPLQLCKEAGISQEEAVELLQMLRDDAQPQQQRAA......ADGVTALDLL....HQEQ 81
Drosophila melanogaster ............MDLRPMILQTCTGRLLSEYQLNL...LRKNNICSLI...DFYDADEKKLHEL..........WAINIQSVRELKKELSLLPKGSVDEG 72
Rhizopus azygosporus ............MSLSQFISSPSLQQKLDNFGFETIEEIKEANLIQLSKDLQLTKEEVEELISVTSKDAKPHKQTMA............DRI.....AQAE 72
Entamoeba histolytica ..............................ML......FIQDKEMNKSNELKIKQM...................KTKTLKELVEE..VKEK 35
logo
Homo sapiens TQGF.IITFCSALDDI...LGGGVPLMKTTEICGAPGVGKTQLCMQLAVDVQIPECFGGVAGEAVFIDTEGSFMVDRVVDLATACIQHLQLIAEKHKGEE 191
Pan troglodytes TQGF.IITFCSALDDI...LGGGVPLMKTTEICGAPGVGKTQLCMQLAVDVQIPECFGGVAGEAVFIDTEGSFMVDRVVDLATACIEHLQLIAEKHKGEE 191
Macaca mulatta TQGF.IITFCSALDDI...LGGGVPLMKTTEICGAPGVGKTQLCMQLAVDVQIPECFGGVAGEAVFIDTEGSFMVDRVVDLATACIQHLQLIAEKHKGEE 189
Tursiops truncatus TQSF.IITFCSALDNI...LGGGIPLTKTTEICGAPGVGKTQLCMQLAVDVQIPECFGGVEGEAVFIDTEGSFMVDRVVDLANACIQHLQLIAGTHMGEE 182
Bos taurus TQNF.IITFCSALDNI...LGGGIPLTKTTEICGAPGVGKTQLCMQLAIDVQIPECFGGVEGEAVFIDTEGSFMVDRVVDLATACIQHLQLIAGTHMEEE 182
Canis lupus f. TQSF.IITFCSALDNI...LGGGIPLTKTTEICGVPGVGKTQLCMQLAVDVQIPECFGGVEGEAVFIDTEGSFMVDRVVDLATACIQHLHLIAGRHMGEE 182
Felis catus TQSF.IITFCSALDNI...LGGGIPLTKTTEICGVPGVGKTQLCMQLAVDVQIPECFGGVEGEAVFIDTEGSFMVDRVVDLATACIQHLHLIAEAHMGEE 182
Rattus norvegicus TQGF.IITFCSALDNI...LGGGIPLMKTTEVCGVPGVGKTQLCMQLAVDVQIPECFGGVAGEAVFIDTEGSFMVDRVVSLATACIQHLHLIAGTHTEEE 182
Mus musculus TQGF.IITFCSALDNI...LGGGIPLMKTTEVCGVPGVGKTQLCMQLAVDVQIPECFGGVAGEAVFIDTEGSFMVDRVVSLATACVRHLHLIAGTHTEEE 182
Gallus gallus TQGF.IITFCSALDDI...LGGGVQLTKITEICGAPGVGKTQLCMQLAVDVQIPECFGGVAGEAVFIDTEGSFMVDRAADIATACVRHCQLIAEAHQEED 182
Xenopus tropicalis SQGF.VITFCSALDEI...LGGGIPVAKITEICGVPGVGKTQLCMQLAVDVQIPECFGGVAGETVFIDTECSFRLERLMDIANACVQHCNLIAQGHQDKD 177
Danio rerio TLGS.IVTFCSGLDDA...IGGGVPVGKTTEICGAPGVGKTQLCMQLAVDVQIPVFFGGLGGKALYIDTEGSFLVQRVADMAEAAVQHCTLLAEDT...E 174
Drosophila melanogaster TPDFNYDTGIEELDKLLDSVEQPFKPGRVWELCGQPGVGKTQLLYTLALNFVWKH.....SQAVLFIDTKREFSCKRIQDMLRAREVDEEASERAMKGIR 167
Rhizopus azygosporus EQGI.SI.SSNNINRL...LDGGVPAYKITEISGESGSGKTQVCMQLAVNVQLKPEEGGVQGECIYIDTEGSFLTSRLIDMVR................ 150
Entamoeba histolytica KVRN.IPTFNQEIDQF...LNGGISLGEITQIVGFPGSGKSQLCMQIACNVQLPEEIGGLNSESIYYDSYSQFCISRVQRMAECICASY.......... 120
logo
Homo sapiens HRKALEDFTLDNILSHIYYFRCRDYTELLAQVYLLPDFLSEHSKVRLVIVDGIAFPFRH..DLDDLSLRTRLLNGLAQQMISLANNHRLAVILTNQM... 286
Pan troglodytes HQKALEDFTLDNILSHIYYFRCRDYTELLAQVYLLPDFLSEHSKVRLVIVDGIAFPFRH..DLDDLSLRTRLLNGLAQQMISLANNHRLAVILTNQM... 286
Macaca mulatta HRKALEDFTLDNILSHIYYFRCRDYTELLAQVYLLPDFLSEHSKVRLVIVDGIAFPFRH..DLDDLSLRTRLLNGLAQQMISLANNHRLAVILTNQM... 284
Tursiops truncatus HPKALQDFTLENILSHIYYFRCRDYTELLAQVYLLPDFLSEHSKVRLVIVDGIAFPFRH..DLDDLSLRTRLLNGLAQQMISLANNHRLAVILTNQM... 277
Bos taurus HPKALQDFTLENILSHIYYFRCRDYTELLAQVYLLSDFLSEHSKVRLVIVDGIAFPFRH..DLDDLSLRTRLLNGLAQQMISLANNHRLAVILTNQM... 277
Canis lupus f. HSKALEDFTLENILSHIYYFRCRDYTELLAQVYLLPDFLSEHSKVRLVIVDGIAFPFRH..DLDDLSLRTRLLNGLAQQMISLANNHRLAVILTNQM... 277
Felis catus HPKALEDFTLENILSHIYYFRCRDYTELLAQVYLLPDFLSEHSKVRLVIVDGIAFPFRH..DLDDLSLRSRLLNGLAQQMISLANNHRLAVLLTNQM... 277
Rattus norvegicus QQKALKDFTLENILSHIYYFRCHDYTELLAQVYLLPDFLSDHSKVQLVIIDGIAFPFRH..DLDDLFLRTRLLNGLAQQLISLANKHRLAVILTNQM... 277
Mus musculus HQKALKDFTLENILSHIYYFRCHDYTELLAQVYLLPDFLSDHPKVQLVIIDGIAFPFRH..DLEDLSLRTRLLNGLAQQMISLANNHRLAVILTNQM... 277
Gallus gallus HLQALETFSLESILSHIYYFRCRDYIELLAQVYLLPDFLSEHSKVRLVVIDGIAFPFRH..DFEDLSLRTRLLNGLAQQLIIIANDHKSAVVLTNQM... 277
Xenopus tropicalis HIKAMQTFTLNEILSQIYYFSCHDYIELLAQINLLPDFLSSHPKVKLVVIDSIAFPFRH..SFEDLSLRTRLLNGFGQQLISLAHNCNLAVVLTNQM... 272
Danio rerio QKGALEELNVEKILSNLFLVRCHDYVKLLAEVYLLPDFLSEHPEVRLVVIDSIAFPFRH..DFEDLSQRTRLLNGLAQQLIQLATQHRVAVVLTNQM... 269
Drosophila melanogaster VVQAATGADINDLLKSF...D........HQLTAETHASMQTKLVLIDSLAACFAFYRGRRMRDVRKSVLTELACKIRKLALRG.VAFVIGNVSFFE 252
Rhizopus azygosporus ......KYDIESVSKGIHLFRVLNHIELIALVRQLPSILKEYTNI..VVTNHV... 195
Entamoeba histolytica ...PEYKLNVKEILEKIHVYQPHDIVSLLCSSLL...SINNKLNKVKVIIIDSIPTFYKK..AMCNDTIRLAAIHRIIQILSIYSNKYYLSVVIVNHL... 209
logo
Homo sapiens .......TTKIDRNQALLV......PAL.......GESWGHAATIRLIFHWDRKQRL.........ATLYKSP.SQKECTVLFQIKPQGFRDTVVTSACS 356
Pan troglodytes .......TTKIDRNQALLV......PAL.......GESWGHAATIRLIFHWDRKQRL.........ATLYKSP.SQKECTVLFQIKPQGFRDTVVTSACS 356
Macaca mulatta .......TTKIDKNQALLV......PAL.......GESWGHAATIRLIFHWDRKQRL.........ATLYKSP.SQKESTVLFQIKPQGFRDTVVTSACS 354
Tursiops truncatus .......TTKIDKNQALLV......PAL.......GESWGHAATIRLIFHWDQKRRL.........ATLYKSP.SQKDATVLFQITPQGFRDAVVATACS 347
Bos taurus .......TTKFDRNQALLV......PAL.......GESWGHAATIRLIFHWDQKQRL.........ATLYKSP.SQKESTVPFQITPQGFRDAIVATAYS 347
Canis lupus f. .......TTKIDRNQALLV......PAL.......GESWGHAATIRLIFHWDQKQRL.........ATLYKSP.SQKESTVLFQITPQGFRDAVVVTACS 347
Felis catus .......TTKIDRNQALLV......PAL.......GESWGHAATIRLIFHWDQNQRL.........ATLCKSP.SQKESTVLFQITPQGFRDAVVAPACS 347
Rattus norvegicus .......TTKIDKNQASLV......PAL.......GESWGHAATIRLIFHWEQKQRF.........ATLYKSP.SQKESTVPFQITPQGFRDAVVTAA.S 346
Mus musculus .......TTKIDKNQALLV......PAL.......GESWGHAATIRLIFHWEQKQRF.........ATLYKSP.SQKESTIPFQITPQGFRDAVVTAA.S 346
Gallus gallus .......TTRFGQNQSMLV......PAL.......GESWGHAATVRLIFHWDNTQRL.........ATLYKSP.SQKESTIPYNITPQGFRDVQPPPVTQ 347
Xenopus tropicalis .......TTKIGPSDSKLV......PAL.......GESWGHASTIRLILHWKSKQRF.........VTLCKSP.SQKKATVPYGITSQGFRDAEIAEPEA 342
Danio rerio .......TTRVSNGQSKLV......PAL.......GESWGHAATQRLILHWEGQRRL.........ASLYKSP.SQMEATVQYQITVQGFRDSPDEPRPT 339
Drosophila melanogaster NNKDSCGDDGEQNGDDEEVTRQQLEPML.......GSYWSSVATLRLSVELPEEEDFTLQDDGLRFIYVISNTYGPDGEHCLLRLITDAGVV......... 336
Rhizopus azygosporus .......T..VNSIDGRWT......PSL.......GPSWGHWCHNRLYLYRKRQFRF.........GYLFKASESSQQIPVQFCIKEDGLGDPDEQEMAL 264
Entamoeba histolytica .......TTKKINSNYTSIDYTQSAPFLTYFLRNGGVIFTSYI.KNSIFLYQIESKY.........KAIIQTTTRLQNGNISFEITEDGFR......... 283
logo
Homo sapiens ....LQTEGSLSTRKRSRDPEEEL... 376
Pan troglodytes ....WQTEGSLSTRKRSRDPEEEL... 376
Macaca mulatta ....LQTEGSLSTRKRSREPEEEL... 374
Tursiops truncatus ....LQTEGSLNIRKRSRDSEEEKESK........D.. 371
Bos taurus ....LQTEGSLNSRKRSRDSEEEQESK........D.. 371
Canis lupus f. ....LQTEGSLNSRKRSRESEEEQESK........D.. 371
Felis catus ....WQTEGSLNSRKRSRE.. 362
Rattus norvegicus ....SQTESSLNFRKRSREPEEEC... 366
Mus musculus ....SQTESSLNFRKRSREPEEEC... 366
Gallus gallus ....NAEGTEMNPRKRPRREEEK.. 366
Xenopus tropicalis ....NALIPASNPRKRPRVDDDV.. 361
Danio rerio FDPSEVSSPSANHSKRPRLEDLS.. 362
Drosophila melanogaster 336
Rhizopus azygosporus FKTEG...EEINNKIESTLEELQLSTSAEVKAEEDWDEIEVMDDVPSESIVIDTKPQSSQPVAHGQKRSRGDKEDSEAEYWSSDIEEDLHFLINMQETF 360
Entamoeba histolytica 283
X non-conserved
X ≥50% conserved
