# Supplementary material for: Rad51 paralogs and the risk of unselected breast cancer: A case-control study
Source: PLoS One. 2020 Jan 6;15(1):e0226976. doi: 10.1371/journal.pone.0226976 (PMC6944361; doi:10.1371/journal.pone.0226976)
Supplement: S1 File — This supporting information file provides the full and detailed description of the RF-based strategy used to analyze simple associations between SNP predictors and BrC (Part A), as well as algorithms used in analysis of epistatic interactions between SNPs and BrC (Part B: pRF; Part C: MB-MDR). (DOCX) [file pone.0226976.s002.docx]

**Part A:** RF-based strategy used to analyze the simple associations between predictors and BrC

The whole strategy used to reliably validate and statistically infer on the ranking of all analyzed predictors with respect to their ability to accurately predict the BrC/control status consisted of three below described steps.

In the first step, the best RF model was searched for based on systematic examination of the impact of three crucial parameters affecting the resultant RF on its ability to precisely predict the BrC/control status: the number of decision trees in the forest (*ntree*), the minimal number of data points in a terminal node of a decision tree (*nodesize*) and the maximum depth to which a tree should be grown (*nodedepth*). All possible combinations of six different levels of *ntree* (50, 100, 150, 200, 250 300) and eleven levels of both the *nodesize* and *nodedepth* (integers from 1 to 10 plus NULL (i.e. no restriction)) were tested, yielding a total number of 726 different RF settings. For each combination of *ntree*, *nodesize* and *nodedepth* a total of 150 RFs were grown using a randomly chosen training dataset (90% of the whole dataset). For each RF the testing prediction error ${}_{i,j,k}\varepsilon$was obtained calculating the number of incorrect predictions in the remaining 10% of the original dataset (testing dataset was used to avoid any possible overlap due to bootstrap method inherent to RF methodology). Given combination of *ntree*, *nodesize* and *nodedepth* was then characterized by mean of the testing prediction error estimate ${}_{i,j,k}\bar{\varepsilon}$obtained as a simple average of such 150 ${}_{i,j,k}\varepsilon$values. This procedure was repeated 10 times, as a result of which, the grand average testing prediction error estimate ${}_{i,j,k}E$was obtained for each combination of *ntree*, *nodesize* and *nodedepth* by averaging the 10 ${}_{i,j,k}\bar{\varepsilon}$estimates:

${}_{i,j,k}E=\frac{1}{10}\sum_{m=1}^{10} {}_{i,j,k}\bar{\varepsilon}_{m}=\frac{1}{10}\sum_{m=1}^{10} \left(\frac{1}{150}\sum_{n=1}^{150} {}_{i,j,k}\varepsilon_{m}^{n}\right)$,

where m ∈ <1;10> is the number of run, n ∈ <1;150> indicates the number of a RF in a given run, and *i, j, k* represent the i^th^, j^th^ and k^th^ level of three crucial RF parameters. The values of *ntree*, *nodesize* and *nodedepth* at which the ${}_{i,j,k}E$estimate reached its minimum were chosen as the RF settings of choice. All other RF parameters were kept as default, including the number of predictors randomly selected as candidates for each node split (*mtry*; set to the square root of the total number of predictors used in the analysis).

In the second step, the combination of *ntree*, *nodesize* and *nodedepth* with the lowest ${}_{i,j,k}E$value was used to grow an RF upon the whole dataset (without splitting it into training and testing subsets). Performance of this RF model was characterized by the classification error obtained based on the whole dataset and the model itself was retained for subsequent RF-based analyses of epistatic interactions, as well. Using this model, all involved predictors were ranked based on their VIMP values. For each predictor, the significance of observed VIMP was estimated based on null distribution of VIMP obtained empirically by permuting the response variable 10,000 times.

Subsequently, in the third step of the strategy, obtained VIMP-based ranking of predictors was validated by means of the bootstrap technique taking advantage of the resampling with replacement inherent to the RF methodology itself. The ranking procedure was bootstrapped 10,000 times, during which the RF ensembles were grown using the same best combination of *ntree*, *nodesize* and *nodedepth* obtained in the first step of the strategy. For each of the 10,000 runs, the VIMP-based ranking of all predictors was obtained, based on which a bootstrap estimate of the distribution of ranks for all predictors was obtained. Finally, all predictors were ranked according to their weighted average ranks and the distribution of ranks of respective predictors were presented in the form of a heatmap.

**Part B:** Random Forest analysis of epistatic interactions

In the procedure, the retained RF model used to elucidate the VIMP-based ranking of predictors (obtained using the original dataset and the best combination of *ntree*, *nodesize* and *nodedepth*; see S1 Text supporting information file) was used.

According to the premise provided by Li et al. in their original report describing the employed algorithm (Li J et al., BioData Mining; 2016;9(1):14), if an interaction between predictors is associated with BrC/control status, its involvement into an RF model predicting such status should greatly improve its prediction power. Equivalently, deleting such interaction would increase the classification error of such a model.

Therefore, the procedure was based on two permutation strategies employed to generate two testing datasets, differing from each other by the mere preservation or deletion of the interaction among the analyzed pair or triplet of predictors. Subsequently, both these permuted datasets were dropped down the retained RF model and classification errors for testing dataset with (*E_1_*) or without (*E_2_*) the interaction among analyzed predictors were calculated (erroneous classification was assumed if the classification resulting from the RF model was different from the actual BrC/control status). Calculation of *E_1_* and *E_2_* classification errors was repeated 20 times for each 2-way and 3-way combination of predictors, based on which the average classification errors$\bar{E}_{1}$and$\bar{E}_{2}$were calculated. Now the subtraction $\Delta E=\bar{E}_{2}-\bar{E}_{1}$, termed the differential error, was used as the measure of how much the RF model prediction power changes when the interaction among selected predictors is deleted from the dataset. If an interaction between predictors contributes greatly to RF model prediction power, deleting such interaction would increase the prediction error of the RF model yielding higher values of ΔE. Therefore, the higher the ΔE, the stronger the association between the interaction and an output (BrC/control status in our study). All possible 2-way and 3-way combinations of predictors were thus ranked according to their calculated ΔE values, with those characterized by the highest ΔE being considered as the top combinations of predictors, interactions among which may be relevant with respect to BrC/control status.

**Part C:** Analysis of epistatic interactions using model-based multifactor dimensionality reduction (MB-MDR)

MB-MDR (Calle ML et al., Stat. Med. 2008;27(30):6532.) [51] is an extension of the original multifactor dimensionality reduction (MBR) algorithm for reduction of the dimensionality of genetic data, thoroughly described elsewhere (Moore JH et al., J. Theor. Biol. 2006;241(2):252; Ritchie M et al., Am. J. Hum. Genet. 2001;69(1):138; Velez DR et al., Genet. Epidemiol. 2007;31(4):306). MB-MDR uses a constructive induction technique to merge multi-locus genotypes into a one-dimensional construct, assigning each analyzed combination of genotypes to either “high-risk”, “low-risk”, or a “no-evidence” (or “non-informative”) category based on possessed genotypes. Then, the new predictive variable with three states (H, L, 0) is tested for association with the risk of outcome (BrC in this study). This method has been shown to be more powerful than most parametric methods in testing higher-order interactions, mainly due to inherent nonlinear nature of epistasis and the implications of multiple comparison testing, colloquially known as „the curse of dimensionality” (Moore JH et al., J. Theor. Biol. 2006;241(2):252).

In our analyses, we used the *mbmdr* package for R obtained from the CRAN repository [54]. For all SNPs additive genetic model was assumed and only second- and third-order SNP combinations were investigated. Logistic regression was used as a link function for association test facilitating the multi-locus genotype-based categorization into H, L or 0 group. In line with default settings, the categorization itself was based on the value and significance of regression coefficient *β* adjusted to age and smoking status: genotype combinations with the *p-*value smaller than 0.10 were considered as “high-risk” or “low-risk” if *β* > 0 or *β* < 0, respectively, while those, for which the *p*-value was above 0.10, were considered as “non-informative”. After merging the multi-locus genotypes of the same risk class, the H and L risk groups were tested for association with BrC versus the remaining two groups by logistic regression (with adjustment to age and smoking status), and respective two Wald statistics – W_H_ and W_L_ (together with regression coefficients *β*_H_ and *β*_L_) – were obtained. Based on W_H_ and W_L_, respective *p* values were calculated (*p*_H_ and *p*_L_) and the minimum of these two values was used as the resulting test statistics of the epistatic effect under investigation. Finally, testing the max{W_H_,W_L_} statistic against the permutational distribution of the Wald statistic implemented in the *mbmdr* package, with 10,000 permutations, was used to correct the obtained *p-*levels for multiple hypothesis testing.
